# Protective effects of melatonin against oxidative stress induced by metabolic disorders in the male reproductive system: a systematic review and meta-analysis of rodent models

**DOI:** 10.3389/fendo.2023.1202560

**Published:** 2023-07-05

**Authors:** Niloofar Dehdari Ebrahimi, Alireza Sadeghi, Moein Ala, Fatemeh Ebrahimi, Sara Pakbaz, Negar Azarpira

**Affiliations:** ^1^ Transplant Research Center, Shiraz University of Medical Sciences, Shiraz, Iran; ^2^ Experimental Medicine Research Center, School of Medicine, Tehran University of Medical Sciences, Tehran, Iran; ^3^ Student Research Committee, Shiraz University of Medical Sciences, Shiraz, Iran; ^4^ Department of Pathology, University of Toronto, Toronto, ON, Canada

**Keywords:** melatonin, animal model, meta-analysis, diabetes, obesity, systematic review, metabolic disorders, thyroid disorders

## Abstract

**Background:**

Male infertility is a multifaceted issue that has gained scientific interest due to its increasing rate. Studies have demonstrated that oxidative stress is involved in male infertility development. Furthermore, metabolic disorders, including obesity, diabetes, hypo- and hyperthyroidism, are risk factors for male infertility, and oxidative stress is believed to contribute to this association. Melatonin, functioning as an oxidative scavenger, may represent a promising therapeutic approach for the prevention and treatment of metabolic disorder-associated male infertility.

**Material and methods:**

We systematically searched three online databases (PubMed, Scopus, and Web of Science) for studies that evaluated the effects of melatonin therapy on metabolic disorders-induce infertility in male rodents. The favorable outcomes were histopathological parameters of testicular tissue, reproductive hormones, and markers of oxidative stress. Then, meta-analyses were done for each outcome. The results are reported as standardized mean difference (Cohen’s d) and 95% confidence interval.

**Results:**

24 studies with 31 outcomes were included. Rats and mice were the subjects. Studies have employed obesity, diabetes, hypothyroidism, hyperthyroidism, hyperlipidemia, and food deprivation as metabolic disorders. To induce these disorders, a high-fat diet, high‐fructose diet, leptin, streptozotocin, alloxan, carbimazole, and levothyroxine were used. The outcomes included histopathologic characteristics (abnormal sperm morphology, apoptotic cells, apoptotic index, Johnsen’s testicular biopsy score, seminiferous epithelial height, tubular basement membrane thickness, tubular diameter, sperm count, and motility), weight-related measurements (absolute epididymis, testis, and body weight, body weight gain, epididymal adipose tissue weight, and relative testis to body weight), hormonal characteristics (androgen receptor expression, serum FSH, LH, and testosterone level), markers of oxidative stress (tissue and serum GPx and MDA activity, tissue CAT, GSH, and SOD activity), and exploratory outcomes (serum HDL, LDL, total cholesterol, triglyceride, and blood glucose level). The overall pooled effect sizes were statistically significant for all histopathological characteristics and some markers of oxidative stress.

**Conclusions:**

Melatonin can reduce damage to male rodents’ gonadal tissue and improve sperm count, motility, and morphology in metabolic diseases. Future clinical studies and randomized controlled trials are needed to evaluate the safety and effectiveness of melatonin for male infertility in patients with metabolic diseases.

## 1 Introduction

Male infertility is a prevailing concern, accounting for no less than half of all instances of infertility (1). Scholarly investigations suggest a varying degree of male factor involvement in this reproductive impairment, with Jafari estimating its prevalence to be at 40.9% (2). Further research indicates a wide-ranging distribution of male-related infertility from 20-70%, with the proportion of infertile males ranging from 2.5 to 12% (3). Metabolic diseases can impair sperm analysis and alter the circulatory levels of sex hormones (4). For instance, it has been shown that patients with metabolic syndrome have significantly decreased sperm count, concentration, normal morphology, progressive motility, and vitality and higher sperm DNA fragmentation index, and increased mitochondrial membrane potential (4). In addition, metabolic syndrome predicts lower serum levels of follicle-stimulating hormone (FSH) and testosterone (4). Similarly, it has been reported that compared with normal subjects, patients with diabetes or obesity have markedly reduced semen volume, sperm count, concentration, progressive motility, and testosterone levels (5). Zhao et al. indicated that compared to euthyroid individuals, male patients with subclinical hypothyroidism have significantly higher DNA fragmentation index in their semen analysis. Even, thyroid-stimulating hormone (TSH) level was significantly associated with DNA fragmentation index in this study (6). Likewise, it has been observed that a longer duration of hypothyroidism can cause a greater extent of harm to male reproductive capacity (7). Systemic inflammation and dysregulation of the hypothalamic-pituitary-gonadal (HPG) axis are involved in the pathogenesis of male infertility in metabolic diseases (8).

Melatonin is mainly known for its function as a regulator of the circadian cycle (9). In response to darkness, melatonin is released by the pineal gland (9). Interestingly, male subjects with night shiftwork, insufficient sleep, and poor sleep quality are more likely to have impaired sperm quality and experience infertility (10). Recent studies have uncovered the role of melatonin on different biological processes other than the circadian cycle (11). Their findings have shown that melatonin has anti-inflammatory and anti-oxidant properties and improves mitochondrial dysfunction (11). Seminal fluid contains melatonin, and spermatozoa possess melatonin receptors (12, 13). In previously published research, urinary 6-sulfatoxymelatonin levels from 52 infertile men were positively correlated with sperm concentration, motility, and morphology (12). Furthermore, 30 minutes of ex-vivo exposure to melatonin improved sperm motility and considerably increased the percentage of motile and progressively motile cells (12). It was found that patients with idiopathic oligoasthenoteratozoospermia have significantly lower levels of serum and seminal plasma melatonin. In addition, a significantly positive correlation was observed between serum melatonin levels and sperm motility (14). Previous studies indicated that melatonin can upregulate several endogenous anti-oxidant enzymes such as superoxide dismutase (SOD) and glutathione peroxidase (GPx) (15). Melatonin may enhance spermatogenesis and germ cell viability by attenuating oxidative stress, inflammatory response, and apoptosis and promoting meiosis (16). Recent animal studies have shown that melatonin may reverse the abnormal alterations of the HPG axis, spermatogenesis, and sperm function induced by metabolic and systemic diseases (17, 18).

Therefore, we aimed to perform a systematic review and meta-analysis to dissect the anti-oxidant roles of melatonin in rodent animal models of metabolic disease-associated male infertility.

## 2 Material and methods

This systematic review and meta-analysis is in line with The Preferred Reporting Items for Systematic reviews and Meta-Analyses (PRISMA) statement (19). This study is part of a series of systematic reviews on the protective effects of melatonin against rodent testicular oxidative stress induced by physical and chemical toxins injuries with previously published protocols (20, 21). We ask if melatonin therapy is able to protect the male testicular tissue against metabolic disorders-induced oxidative stress in rodents and try to quantify the effect.

### 2.1 Data sources and search

Three databases were systematically searched for related studies (PubMed, Scopus, and Web of Science). Two reviewers (NDE and AS) searched the databases for records that were published from January 1, 1970, until September 9, 2022, using “melatonin”, “testicular histopathology”, and their related keywords. Thereafter manual citation searching was done based on reference lists of the included studies. The search strategy was not restricted based on the language and is provided in **Supplementary material 1**.

### 2.2 Study selection and eligibility criteria

To begin, the electronic removal of duplicate records was carried out. Subsequently, two reviewers (NDE and AS) independently utilized the Rayyan online tool for managing systematic reviews to screen the records by titles and abstracts (22). Then, full-text screening of the relevant records was conducted to meet the eligibility criteria. Disagreements were resolved through discussions. Studies that satisfied the following requirements were included: (1) controlled animal studies, (2) at least one intervention group receiving a melatonin regimen, (3) rodents exposed to metabolic disorders (such as diabetes, obesity, dyslipidemia, and hypo- and hyperthyroidism) resulting in oxidative stress to testicular tissue, (4) reported hallmarks of testicular tissue (such as histopathologic, biochemical, and sperm analyses), and (5) at least one control group with comparable stress.

Exclusion criteria consisted of the following: (1) non-rodent animals exposed to other types of stresses (such as physical, chemotherapy, toxins, and heavy metals), (2) in-vitro and ex-vivo studies, (3) combination therapy using melatonin with other drugs, (4) failed to report relevant outcomes, (5) healthy controls without metabolic disorders-induced stress, and (6) treatment involving derivatives of melatonin. Additionally, reviews, letters, human trials, and non-peer-reviewed publications were excluded from the review.

### 2.3 Data extraction and risk of bias assessment

Two independent reviewers (NDE and FE) extracted the data into Excel spreadsheets. Controversies were resolved by the involvement of a third author (AS). One reviewer (NDE) cleaned the data to become suitable for analyses. The data were extracted using the following variables: (1) study characteristics (first author and publication year), (2) rodent characteristics (age, species, type of stress, and sample size), (3) melatonin (dosing, duration of treatment, timing, and route of administration), (4) tissue, plasma, and histopathological indices, and (5) time of outcome assessment.

The risk of bias in the studies was evaluated by utilization of the SYRCLE tool for animal intervention studies (23). Each article was independently reviewed by two reviewers (NDE and FE), who classified them as high, low, or unclear for each bias domain. In case of disagreements, a consensus was reached by the involvement of a third reviewer (AS).

### 2.4 Data synthesis and statistical analysis

Meta-analyses were done by a single reviewer (AS) using Stata MP Version 16 (StataCorp, College Station, TX, USA). Two (NDE and MA) independent reviewers supervised the meta-analyses. Under the random effect model (DerSimonian-Laird), Standardized Mean Difference (SMD, Cohen’s d) was utilized as the effect measure and a less than 0.05 p-value was considered statistically significant. The residual between-study heterogeneity was examined using I^2^ and p-value. An I^2^ value of less than 25% was considered low heterogeneity, between 25% and 50% was considered moderate heterogeneity, and greater than 50% was considered substantial heterogeneity (24). For missing data, reviewers contacted the authors *via* available emails and waited at least one month to receive any responses. If missing data were crucial, the study was removed from the analysis. In the case of common control arms, intervention arms were combined using Cochrane’s formula to avoid overcalculations (25). When minimum, first quartile, median, third quartile, and maximum were the only available statistics, mean and standard deviation was estimated utilizing methods outlined in prior research (26, 27).

To explore the sources of heterogeneity, subgroup analyses were done. Subgroup analyses were conducted if at least two separate study arms were available for each subgroup. Also, for outcomes with more than ten observations, publication bias was evaluated using funnel plots and Egger’s regression test.

## 3 Results

### 3.1 Search results

**Figure 1:**
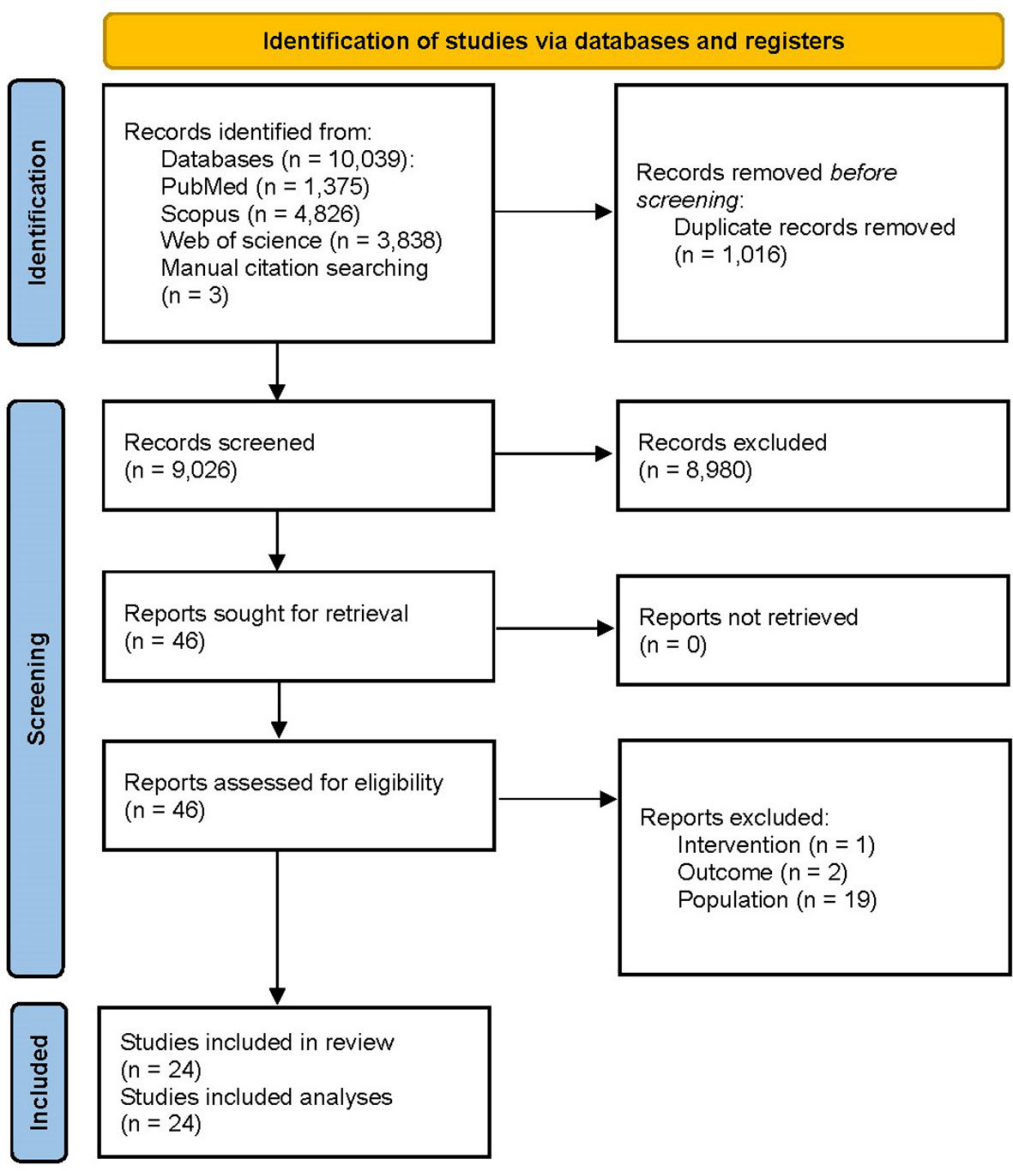
PRISMA flow diagram illustrating the process of selection of the studies.

**Figure 1** summarizes the search results. The systematic database searching resulted in records 10,039 (PubMed: 1,375, Scopus: 4,826, and Web of Science: 3,838) and manual citation searching had three additional results. Using automatic duplicate detection techniques, 1,016 records were removed initially. 9,026 records were screened based on their titles and abstracts which resulted in 46 potentially eligible studies, all of which were successfully retrieved for full-texts. 22 articles were excluded at this stage due to ineligible outcome (n= 2), population (n= 19), and intervention (n= 1). Finally, a total of 24 articles were included in the systematic review and meta-analysis.

### 3.2 Study characteristics

The included studies were published between 2005 and 2022 and all of them were in English. The rodents included in the studies comprised mice (n=3) (28–30) and rats (n=21) (17, 18, 31–49). Across the included studies, rodents were subjected to induction of diabetes (n=11) (18, 28, 31, 34, 35, 38–41, 46, 48), obesity (n=8) (17, 30, 32, 33, 36, 37, 42, 49), hyperthyroidism (n=3) (43, 44, 47) and hypothyroidism (n=1) (44), hyperlipidemia (n=1) (29), and food deprivation (n=1) (45). To induce diabetes, streptozotocin (18, 28, 34, 35, 38–40, 46, 48) and alloxan (31, 41) were used. To induce obesity, high-fat diet (17, 29, 30, 37, 42, 49), high-fructose diet (36), and leptin (32, 33) were used. To induce hyper- and hypothyroidism, levothyroxine (43, 44, 47) and carbimazole (44) were employed, respectively. To induce hyperlipidemia and food deprivation, high-fat diet (17, 29, 30, 37, 49) and one-third of the normal daily food ration (45) were used, respectively. Melatonin was administered orally (n=14) (17, 18, 28–30, 32–34, 36, 39, 41, 44, 46, 49) and intraperitoneally (n=10) (31, 35, 37, 38, 40, 42, 43, 45, 47, 48). All of the studies have administered melatonin after the induction of injuries. **Table 1** presents the study characteristics. The detailed characteristics of the injury induction and melatonin therapy are presented in **Supplementary Material 2**.

**Table 1:** General characteristics of the included studies.

Study name	Rodents	Intervention/control size in each arm	Age of subjects (days)	Mechanism of injury induction	Type of injury	Route of melatonin therapy
**Du 2018 (** 28 **)**	Mice	6/6	NA	Streptozotocin	Diabetes	Oral
**Guneli 2008 (** 40 **)**	Rats	10/10	180-240	Streptozotocin	Diabetes	IP
**Sahan 2020 (** 48 **)**	Rats	10/8	NA	Streptozotocin	Diabetes	IP
**Oliveira 2015 (** 46 **)**	Rats	6/6	5	Streptozotocin	Diabetes	Oral
**Saidi 2022 (** 49 **)**	Rats	6/6	NA	High-fat diet	Obesity	Oral
**Akman 2015 (** 31 **)**	Rats	6/6	56	Alloxan	Diabetes	IP
**Zhang 2012 (** 29 **)**	Mice	6/6	28	High-fat diet	Hyperlipidemia	Oral
**Khalil 2021 (** 42 **)**	Rats	8/8	NA	High-fat diet	Obesity	IP
**Mohamed 2017 (** 44 **)**	Rats	10/10	NA	Thyroxin Carbimazole	Hyperthyroidism Hypothyroidism	Oral
**Oladele 2021 (** 17 **)**	Rats	6/6	NA	High-fat diet	Obesity	Oral
**Mogulkoc 2005 (** 43 **)**	Rats	10/10	56	Levothyroxine	Hyperthyroidism	IP
**Atilgan 2013 (** 37 **)**	Rats	7/7	150-180	High-fat diet	Obesity	IP
**Aslankoc 2019 (** 36 **)**	Rats	12/12	84-112	High‐fructose diet	Obesity	Oral
**Nasiraei-Moghadam 2015 (** 45 **)**	Rats	6/6	NA	One-third of the normal daily food ration	Food deprivation	IP
**Chen 2019 (** 30 **)**	Mice	8/8	49	High-fat diet	Obesity	Oral
**Almabhouh 2016 (** 32 **)**	Rats	6/6	84	Leptin	Obesity	Oral
**Almabhouh 2018 (** 33 **)**	Rats	6/6	84	Leptin	Obesity	Oral
**Alves 2020 (** 34 **)**	Rats	10/10	65	Streptozotocin	Diabetes	Oral
**Ramadan 2020 (** 47 **)**	Rats	15/15	NA	Levothyroxine	Hyperthyroidism	IP
**Gobbo 2015 (** 39 **)**	Rats	10/10	NA	Streptozotocin	Diabetes	Oral
**Armagan 2006 (** 35 **)**	Rats	9/8	77	Streptozotocin	Diabetes	IP
**Hassen 2007 (** 41 **)**	Rats	10/10	NA	Alloxan	Diabetes	Oral
**Da Costa 2016 (** 18 **)**	Rats	15/15	28	Streptozotocin	Diabetes	Oral
**Buzkurt 2019 (** 38 **)**	Rats	6/6	180-240	Streptozotocin	Diabetes	IP

NA, Not available; IP, Intraperitoneal.

### 3.3 Outcomes

**Figure 2:**
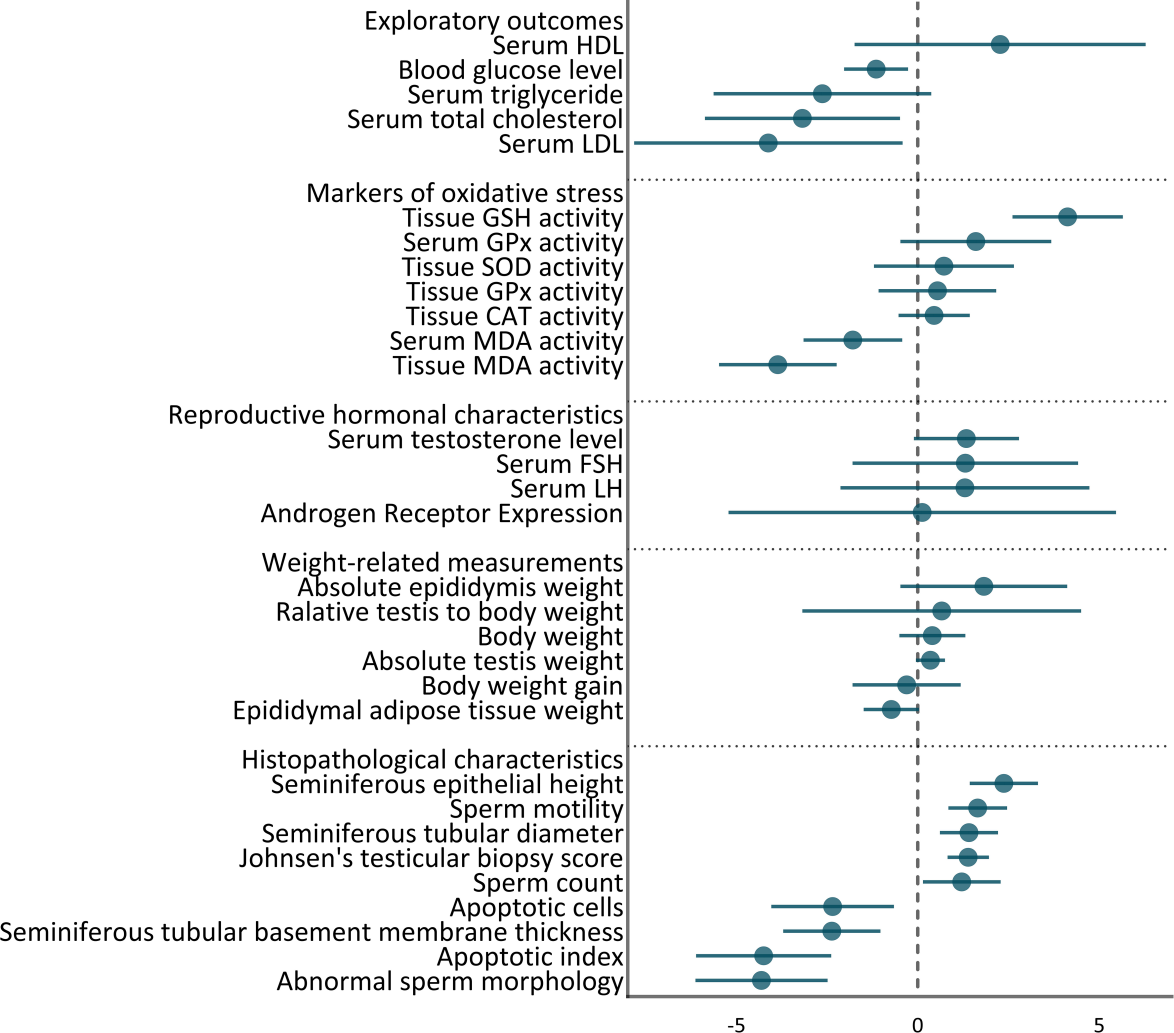
Summary of overall pooled effects sizes for each outcome.

31 outcomes were pooled from the included studies. These outcomes included histopathologic characteristics (abnormal sperm morphology, apoptotic cells, apoptotic index, Johnsen’s testicular biopsy score, seminiferous epithelial height, tubular diameter, tubular basement membrane thickness, sperm count, and motility), weights (absolute epididymis, testis, and body weight, body weight gain, epididymal adipose tissue weight, and relative testis to body weight), hormonal characteristics (androgen receptor expression, serum FSH, LH, testosterone levels), markers of oxidative stress (tissue and serum GPx, andMDA activity, tissue CAT, GSH, and SOD activity), and exploratory outcomes (serum HDL, LDL, total cholesterol, triglyceride, and blood glucose level). The pooled effect sizes are summarized in **Figure 2**.

#### 3.3.1 Histopathological characteristics

**Figure 3:**
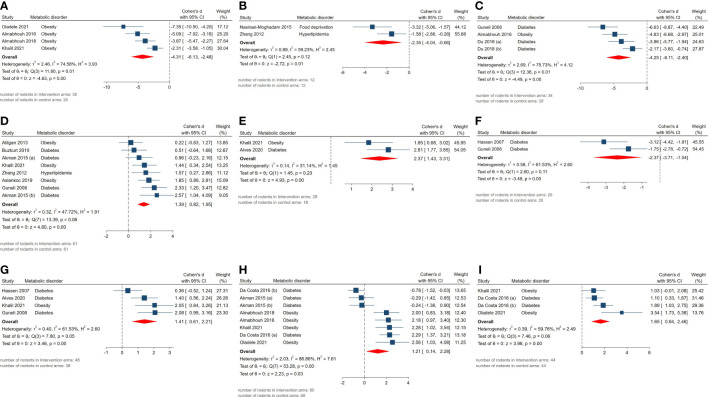
Forest plots for the overall pooled effects sizes of histopathological parameters including **(A)** abnormal sperm morphology, **(B)** apoptotic cells, **(C)** apoptotic index, **(D)** Johnsen’s testicular biopsy score, **(E)** seminiferous epithelial height, **(F)** seminiferous tubular basement membrane thickness, **(G)** seminiferous tubular diameter, **(H)** sperm count, **(I)** sperm motility.

Statistically significant SMDs were observed among histopathological parameters including abnormal sperm morphology (SMD = -4.31, 95% CI: -6.13 to -2.49, p-value <0.01), apoptotic cells (SMD = -2.35, 95% CI: -4.04 to -0.66, p-value 0.01), apoptotic index (SMD = -4.25, 95% CI: -6.11 to -2.39, p-value <0.01), Johnsen’s testicular biopsy score (SMD = 1.39, 95% CI: 0.82 to 1.96, p-value <0.01), seminiferous epithelial height (SMD = 2.37, 95% CI: 1.43 to 3.31, p-value <0.01), tubular diameter (SMD = 1.41, 95% CI: 0.61 to 2.21, p-value <0.01), tubular basement membrane thickness (SMD = -2.37, 95% CI: -3.71 to -1.03, p-value <0.01), sperm count (SMD = 1.21, 95% CI: 0.14 to 2.28, p-value 0.03), and motility (SMD = 1.65, 95% CI: 0.84 to 2.46, p-value <0.01). Residual between-study heterogeneity for each outcome was: abnormal sperm morphology (I^2 =^ 74.58% and p-value for Q test 0.01), apoptotic cells (I^2 =^ 59.23% and p-value for Q test 0.12), apoptotic index (I^2 =^ 75.73% and p-value for Q test 0.01), Johnsen’s testicular biopsy score (I^2 =^ 47.72% and p-value for Q test 0.06), seminiferous epithelial height (I^2 =^ 31.14% and p-value for Q test 0.23), tubular diameter (I^2 =^ 61.53% and p-value for Q test 0.05), tubular basement membrane thickness (I^2 =^ 61.53% and p-value for Q test 0.11), sperm count (I^2 =^ 86.86% and p-value for Q test <0.01), and motility (I^2 =^ 59.76% and p-value for Q test 0.06). The forest plots presenting the analyses on histopathologic parameters are presented in **Figure 3**.

#### 3.3.2 Weight-related measurements

**Figure 4:**
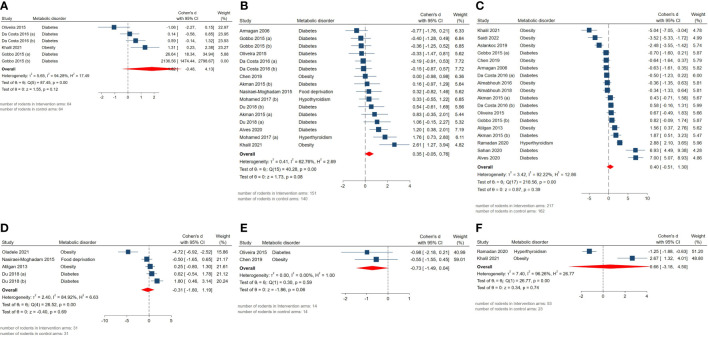
Forest plots for the overall pooled effects sizes of genital weights including **(A)** absolute epididymis weight, **(B)** absolute testis weight, **(C)** body weight, **(D)** body weight gain, **(E)** epididymal adipose tissue weight, **(F)** relative testis to body weight.

Effect measures for absolute epididymis weight (SMD = 1.82, 95% CI: -0.48 to 4.12, p-value 0.12), absolute testis weight (SMD = 0.35, 95% CI: -0.05 to 0.75, p-value 0.08), body weight (SMD = 0.4, 95% CI: -0.51 to 1.31, p-value 0.39), body weight gain (SMD = -0.31, 95% CI: -1.8 to 1.18, p-value 0.69), epididymal adipose tissue weight (SMD = -0.73, 95% CI: -1.49 to 0.03, p-value 0.06), and relative testis to body weight (SMD = 0.66, 95% CI: -3.18 to 4.5, p-value 0.74) were not statistically significant. Although, high residual between-study heterogeneity was found across these outcomes: absolute epididymis weight (I^2 =^ 94.28% and p-value for Q test <0.01), absolute testis weight (I^2 =^ 62.76% and p-value for Q test <0.01), body weight (I^2 =^ 92.22% and p-value for Q test <0.01), body weight gain (I^2 =^ 84.92% and p-value for Q test <0.01), epididymal adipose tissue weight (I^2 =^ 0% and p-value for Q test 0.59), and relative testis to body weight (I^2 =^ 96.26% and p-value for Q test <0.01). The forest plots presenting the analyses on histopathologic parameters are presented in **Figure 4**.

#### 3.3.3 Reproductive hormonal characteristics

**Figure 5:**
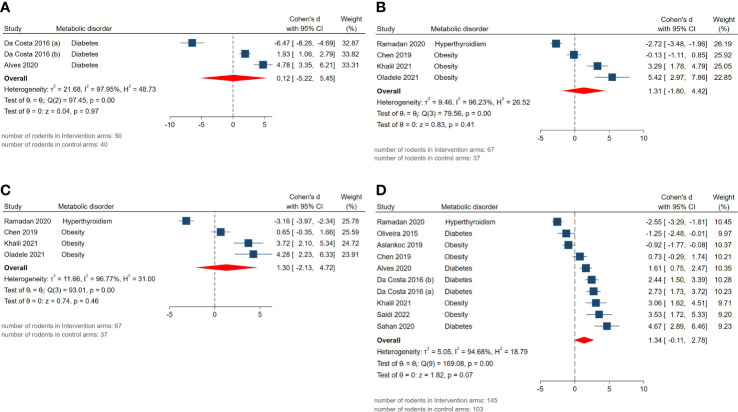
Forest plots for the overall pooled effects sizes of reproductive hormones including **(A)** androgen receptor expression, **(B)** serum FSH, **(C)** LH, **(D)** testosterone level.

The pooled SMDs for reproductive hormone outcomes were not statistically significant: androgen receptor expression (SMD = 0.12, 95% CI: -5.22 to 5.46, p-value 0.97), serum FSH (SMD = 1.31, 95% CI: -1.8 to 4.42, p-value 0.41), LH (SMD = 1.3, 95% CI: -2.13 to 4.73, p-value 0.46), testosterone level (SMD = 1.34, 95% CI: -0.11 to 2.79, p-value 0.07). Also, considerably high residual between-study heterogeneity was found among these outcomes: androgen receptor expression (I^2 =^ 97.95% and p-value for Q test <0.01), serum FSH (I^2 =^ 96.23% and p-value for Q test <0.01), LH (I^2 =^ 96.77% and p-value for Q test <0.01), testosterone level (I^2 =^ 94.68% and p-value for Q test <0.01). The forest plots presenting the analyses on histopathologic parameters are presented in **Figure 5**.

#### 3.3.4 Markers of oxidative stress

**Figure 6:**
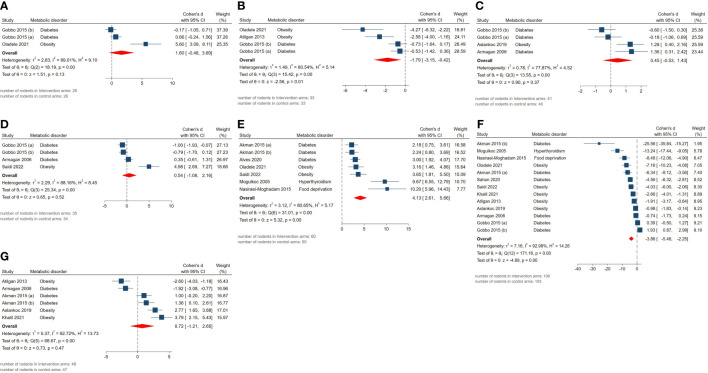
Forest plots for the overall pooled effects sizes of testicular tissue’s oxidative markers including **(A)** serum GPx activity, **(B)** serum MDA activity, **(C)** tissue CAT activity, **(D)** tissue GPx activity, **(E)** tissue GSH activity, **(F)** tissue MDA activity, **(G)** tissue SOD activity.

The pooled SMDs for markers of oxidative stress were as follows: tissue (SMD = 0.54, 95% CI: -1.08 to 2.16, p-value 0.52) and serum GPx activity (SMD = 1.6, 95% CI: -0.48 to 3.68, p-value 0.13), tissue (SMD = -3.86, 95% CI: -5.48 to -2.24, p-value <0.01) and serum MDA activity (SMD = -1.79, 95% CI: -3.15 to -0.43, p-value 0.01), tissue CAT activity (SMD = 0.45, 95% CI: -0.53 to 1.43, p-value 0.37), tissue GSH activity (SMD = 4.13, 95% CI: 2.61 to 5.65, p-value <0.01), tissue SOD activity (SMD = 0.72, 95% CI: -1.21 to 2.65, p-value 0.47). Also, between-study heterogeneity was high across these outcomes: tissue (I^2 =^ 88.16% and p-value for Q test <0.01) and serum GPx activity (I^2 =^ 89.01% and p-value for Q test <0.01), tissue (I^2 =^ 92.99% and p-value for Q test <0.01) and serum MDA activity (I^2 =^ 80.54% and p-value for Q test <0.01), tissue CAT activity (I^2 =^ 77.87% and p-value for Q test <0.01), tissue GSH activity (I^2 =^ 80.65% and p-value for Q test <0.01), tissue SOD activity (I^2 =^ 92.72% and p-value for Q test <0.01). The forest plots presenting the analyses on histopathologic parameters are presented in **Figure 6**.

#### 3.3.5 Exploratory outcomes

**Figure 7:**
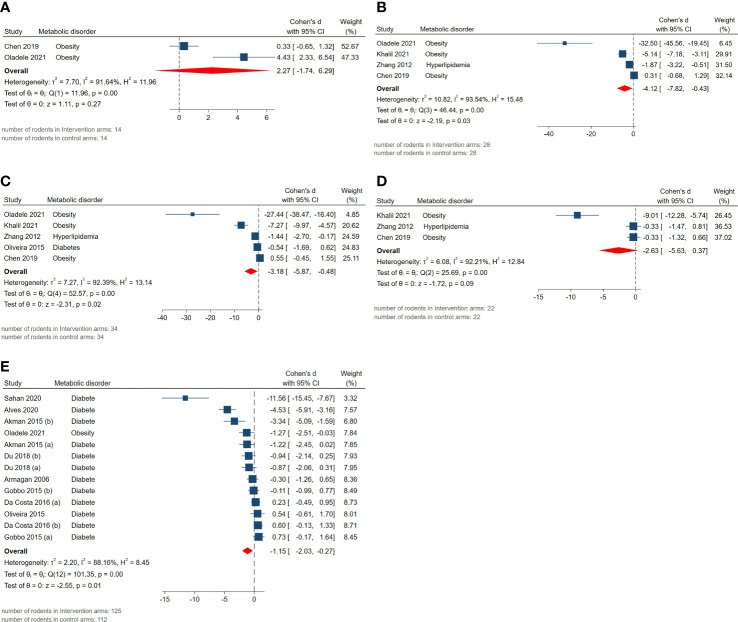
Forest plots for the overall pooled effects sizes of exploratory outcomes including **(A)** serum HDL, **(B)** serum LDL, **(C)** serum total cholesterol, **(D)** serum triglyceride, **(E)** blood glucose level.

The pooled SMDs for exploratory outcomes were serum HDL (SMD = 2.27, 95% CI: -1.74 to 6.28, p-value 0.27), LDL (SMD = -4.12, 95% CI: -7.82 to -0.42, p-value 0.03), total cholesterol (SMD = -3.18, 95% CI: -5.87 to -0.49, p-value 0.02), triglyceride (SMD = -2.63, 95% CI: -5.63 to 0.37, p-value 0.09), blood glucose levels (SMD = -1.15, 95% CI: -2.03 to -0.27, p-value 0.01). High between-study heterogeneity was observed for these outcomes: serum HDL (I^2 =^ 91.64% and p-value for Q test <0.01), LDL (I^2 =^ 93.54% and p-value for Q test <0.01), total cholesterol (I^2 =^ 92.39% and p-value for Q test <0.01), triglyceride (I^2 =^ 92.21% and p-value for Q test <0.01), blood glucose level (I^2 =^ 88.16% and p-value for Q test <0.01). The forest plots presenting the analyses on histopathologic parameters are presented in **Figure 7**.

### 3.4 Risk of bias assessment, sensitivity analysis, and small-study effect

**Figure 8:**
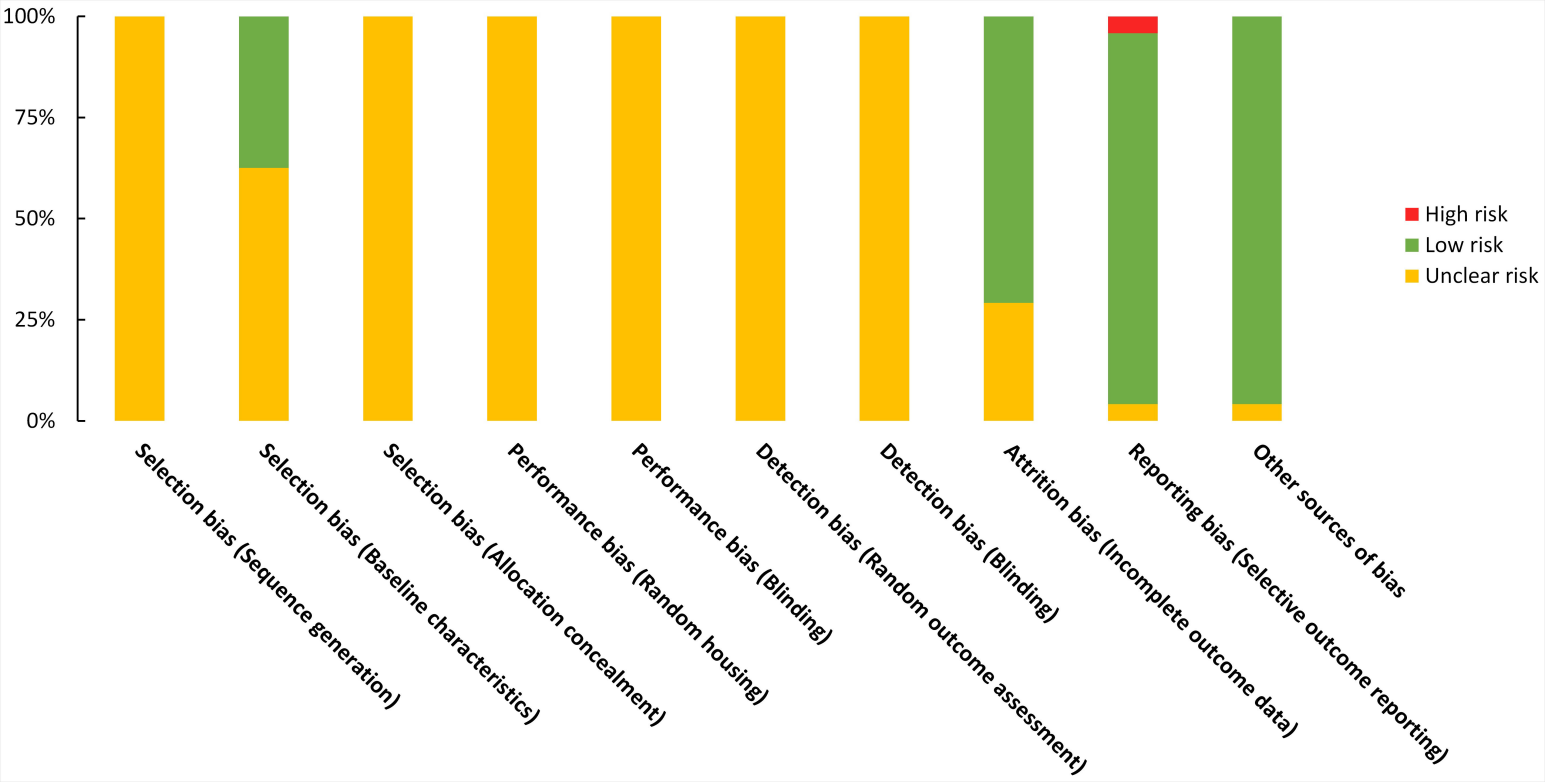
Risk of bias graph on judgements about each risk of bias item presented as proportions across all included studies (n=24).

For each domain of the SYRCLE tool for assessment of the risk of bias in animal studies, studies scored 1 if were low risk. The studies’ overall scores were between 2 and 4. Studies suffered from unclear risk of bias in selection, performance, and detection bias. On the other hand, studies were mostly low risk for bias in attrition and reporting domains. Detailed results of the risk of bias assessment are presented in **Figure 8** and **Supplementary material 3**.

Except for outcomes with a few available studies, in which it is expected that pooled effect size shows low robustness, the leave-one-out meta-analysis demonstrates that the overall effect sizes closely align with the vertical line representing the overall effect size. Additionally, the confidence interval lines of these effect sizes intersect with the vertical red line encompassing all the studies, indicating that no single study has a significant impact on the results of our meta-analysis. The results of the leave-one-out meta-analyses are presented in **Supplementary material 4**.

Using funnel plots and Egger’s regression test, publication bias was assessed and the results are provided in **Supplementary material 5**. Four outcomes were eligible for assessment: absolute testis and body weights, serum testosterone level, and tissue MDA activity, of which, the two formers were asymmetrically scattered on the funnel plots, suggesting the possibility of the small-study effect. Although, due to the presence of high heterogeneity, interpretations should be made with caution.

### 3.5 Subgroup analyses

To explore the heterogeneity, subgroup analyses were done. The results are shown in **Table 2**. 17 studies were eligible for subgroup analyses based on the mechanism of injury induction, melatonin cumulative dose, route of melatonin administration, type of injury, and rodent species. For each outcome, studies were categorized based on the melatonin cumulative dose pertaining to the median value of cumulative doses in that outcome.

**Table 2:** Subgroup analyses.

Outcome	Subgroup		Number of studies	Pooled SMD [95%CI]	I^2^	P value for group differences
**Abnormal sperm morphology**	Mechanism of injury induction	High-fat diet	2	-4.62 [-9.54, 0.31]	88.18	0.923
		Leptin	2	-4.37 [-5.60, -3.14]	_	
	Melatonin cumulative dose	≤385 mg/kg	2	-4.61 [-9.53, 0.30]	88.18	0.923
		>385 mg/kg	2	-4.36 [-5.59, -3.13]	0	
**Absolute testis weight**	Mechanism of injury induction	Alloxan	2	0.48 [-0.34, 1.30]	_	0.083
		High-fat diet	2	1.26 [-1.29, 3.82]	89.52	
		Streptozotocin	9	0.04 [-0.40, 0.48]	51.67	
	Type of injury	Diabetes	11	0.10 [-0.29, 0.49]	45.29	0.056
		Obesity	2	1.26 [-1.29, 3.82]	89.52	
	Route of melatonin therapy	IP	5	0.58 [-0.47, 1.62]	76	0.603
		Oral	11	0.28 [-0.15, 0.70]	56.86	
	Melatonin cumulative dose	≤77 mg/kg	8	0.08 [-0.3, 0.53]	50.58	0.159
		>77 mg/kg	8	0.65 [0.00, 1.31]	66.55	
**Body weight**	Mechanism of injury induction	Alloxan	2	1.10 [-0.30, 2.51]	60.19	<0.001
		High-fat diet	4	-1.81 [-4.42, 0.81]	92.69	
		Leptin	2	-0.35 [-1.05, 0.35]	_	
		Streptozotocin	8	1.44 [0.15, 2.73]	92.18	
	Type of injury	Diabetes	10	1.35 [0.27, 2.43]	90.42	<0.001
		Obesity	7	-1.40 [-2.73, -0.07]	88.44	
	Route of melatonin therapy	IP	7	1.10 [-0.73, 2.92]	93.52	0.284
		Oral	11	-0.03 [-0.98, 0.92]	89.82	
	Melatonin cumulative dose	≤175 mg/kg	9	0.56 [-0.26, 1.38]	86.25	0.799
		>175 mg/kg	9	0.29 [-1.60, 2.19]	94.62	
**Body weight gain**	Mechanism of injury induction	High-fat diet	2	-2.14 [-7.01, 2.73]	93.73	0.083
		Streptozotocin	2	1.16 [0.01, 2.32]	41.4	
	Type of injury	Diabetes	2	1.16 [0.01, 2.32]	41.4	0.083
		Obesity	2	-2.14 [-7.01, 2.73]	93.73	
	Route of melatonin therapy	IP	2	-0.09 [-0.87, 0.68]	_	0.743
		Oral	3	-0.62 [-3.68, 2.44]	92.03	
	Melatonin cumulative dose	≤140 mg/kg	3	0.12 [-0.51, 0.77]	0	0.642
		>140 mg/kg	2	-1.39 [-7.79, 4.99]	95.93	
**Johnsen’s testicular biopsy score**	Mechanism of injury induction	Alloxan	2	1.69 [0.13, 3.25]	61.8	0.647
		High-fat diet	3	1.03 [0.16, 1.90]	42.48	
		Streptozotocin	2	1.42 [-0.37, 3.21]	79.58	
	Type of injury	Diabetes	4	1.54 [0.56, 2.53]	60.35	0.845
		Obesity	3	1.18 [0.21, 2.16]	62.26	
	Route of melatonin therapy	IP	6	1.28 [0.54, 2.02]	58.15	0.387
		Oral	2	1.75 [0.98, 2.52]	_	
	Melatonin cumulative dose	≤145 mg/kg	4	1.13 [0.15, 2.12]	65.66	0.390
		>145 mg/kg	4	1.63 [1.06, 2.20]	0	
**Seminiferous tubular diameter**	Route of melatonin therapy	IP	2	2.06 [1.25, 2.87]	_	0.077
		Oral	2	0.89 [-0.13, 1.91]	64.26	
	Melatonin cumulative dose	≤152.5 mg/kg	2	1.18 [-0.49, 2.86]	82.61	0.649
		>152.5 mg/kg	2	1.41 [0.61, 2.20]	0	
**Serum FSH**	Route of melatonin therapy	IP	2	0.25 [-5.64, 6.13]	97.96	0.577
		Oral	2	2.53 [-2.9, 7.96]	94.13	
	Melatonin cumulative dose	≤210 mg/kg	2	-1.44 [-3.98, 1.09]	94.04	0.001
		>210 mg/kg	2	4.12 [2.08, 6.16]	52.82	
**Serum LDL**	Route of melatonin therapy	Oral	3	-3.52 [-7.77, 0.73]	93.54	0.499
	Melatonin cumulative dose	≤210 mg/kg	2	-0.72 [-2.85, 1.40]	84.51	0.207
		>210 mg/kg	2	-18.03 [-44.80, 8.73]	93.93	
**Serum LH**	Route of melatonin therapy	IP	2	0.24 [-6.49, 6.98]	98.19	0.587
		Oral	2	2.35 [-1.20, 5.90]	89.67	
	Melatonin cumulative dose	≤210 mg/kg	2	-1.26 [-4.99, 2.46]	97	0.010
		>210 mg/kg	2	3.93 [2.66, 5.20]	0	
**Serum MDA activity**	Mechanism of injury induction	High-fat diet	2	-3.26 [-4.88, -1.63]	43.47	0.003
		Streptozotocin	2	-0.63 [-1.27, 0.00]	_	
	Type of injury	Diabetes	2	-0.63 [-1.27, 0.00]	_	0.003
		Obesity	2	-3.26 [-4.88, -1.63]	43.47	
	Melatonin cumulative dose	≤25.56 mg/kg	2	-0.63 [-1.26, 0.00]	0	0.003
		>25.56 mg/kg	2	-3.25 [-4.87, -1.63]	43.47	
**Serum testosterone level**	Mechanism of injury induction	High-fat diet	3	2.34 [0.47, 4.21]	81.4	<0.001
		Streptozotocin	5	1.98 [0.46, 3.50]	89.59	
	Type of injury	Diabetes	5	1.98 [0.46, 3.50]	89.59	<0.001
		Obesity	4	1.50 [-0.51, 3.50]	91.14	
	Route of melatonin therapy	IP	3	1.68 [-3.14, 6.50]	97.64	0.856
		Oral	7	1.22 [-0.04, 2.48]	90.45	
	Melatonin cumulative dose	≤142 mg/kg	5	0.41 [-1.79, 2.62]	96.2	0.215
		>142 mg/kg	5	2.29 [0.31, 4.26]	92.38	
**Sperm count**	Mechanism of injury induction	Alloxan	2	-0.26 [-1.07, 0.54]	_	<0.001
		High-fat diet	2	2.39 [1.42, 3.36]	_	
		Leptin	2	2.09 [1.25, 2.94]	_	
		Streptozotocin	2	0.75 [-2.26, 3.76]	96.13	
	Type of injury	Diabetes	4	0.25 [-1.22, 1.72]	89.2	0.016
		Obesity	4	2.22 [1.58, 2.86]	_	
	Route of melatonin therapy	IP	3	0.56 [-1.04, 2.17]	82.05	0.351
		Oral	5	1.61 [0.11, 3.10]	89.96	
	Melatonin cumulative dose	≤343 mg/kg	4	0.90 [-0.84, 2.64]	91.37	0.555
		>343 mg/kg	4	1.54 [0.32, 2.75]	75.83	
**Sperm motility**	Mechanism of injury induction	High-fat diet	2	2.18 [-0.28, 4.62]	81.9	0.591
		Streptozotocin	2	1.47 [0.70, 2.24]	43.98	
	Type of injury	Diabetes	2	1.47 [0.70, 2.24]	43.98	0.591
		Obesity	2	2.18 [-0.28, 4.62]	81.9	
	Melatonin cumulative dose	≤168.59 mg/kg	2	1.47 [0.70, 2.23]	43.98	0.591
		>168.59 mg/kg	2	2.17 [-0.27, 4.62]	81.9	
**Tissue GSH activity**	Mechanism of injury induction	Alloxan	2	2.21 [1.20, 3.23]	_	<0.001
		High-fat diet	2	3.39 [2.14, 4.64]	_	
	Type of injury	Diabetes	3	2.58 [1.85, 3.32]	_	<0.001
		Obesity	2	3.39 [2.14, 4.64]	_	
	Route of melatonin therapy	IP	4	5.60 [2.18, 9.01]	90.2	0.174
		Oral	3	3.16 [2.35, 3.98]	_	
	Melatonin cumulative dose	≤200 mg/kg	4	5.67 [2.64, 8.70]	89.56	0.086
		>200 mg/kg	3	2.89 [1.95, 3.84]	0	
**Tissue MDA activity**	Mechanism of injury induction	Alloxan	2	-15.29 [-34.07, 3.50]	91.99	<0.001
		High-fat diet	4	-3.50 [-5.18, -1.82]	72.98	
		Streptozotocin	4	-0.64 [-2.69, 1.41]	92.78	
	Type of injury	Diabetes	6	-2.74 [-5.22, -0.27]	94.05	<0.001
		Obesity	5	-2.90 [-4.42, -1.39]	81.24	
	Route of melatonin therapy	IP	8	-5.71 [-8.06, -3.36]	91.34	0.009
		Oral	5	-1.56 [-3.60, 0.48]	92.67	
	Melatonin cumulative dose	≤336 mg/kg	8	-4.39 [-6.98, -1.80]	94.56	0.388
		>336 mg/kg	5	-2.95 [-4.97, -0.92]	89.77	
**Tissue SOD activity**	Mechanism of injury induction	Alloxan	2	1.17 [0.30, 2.04]	_	<0.001
		High-fat diet	2	0.58 [-5.69, 6.85]	97	
	Type of injury	Diabetes	3	0.13 [-1.94, 2.21]	88.81	0.589
		Obesity	3	1.31 [-2.44, 5.07]	95.48	
	Melatonin cumulative dose	≤385 mg/kg	3	0.71 [-2.66, 4.09]	94.19	0.992
		>385 mg/kg	3	0.73 [-2.06, 3.53]	94.13	
**Blood glucose level**	Mechanism of injury induction	Alloxan	2	-2.18 [-4.25, -0.11]	73.51	0.572
		High-fat diet	1	-1.27 [-2.511, -0.03]	.	
		Streptozotocin	10	-0.94 [-1.95, 0.06]	89.46	
	Rodent	Mice	2	-0.90 [-1.74, -0.06]	0	0.625
		Rat	11	-1.23 [-2.27, -0.20]	89.98	
	Route of melatonin therapy	Intraperitoneal	4	-3.39 [-6.08, -0.69]	91.68	0.049
		Oral	9	-0.54 [-1.41, 0.32]	85.57	
	Melatonin cumulative dose	≤150 mg/kg	7	0.08 [-0.40, 0.57]	47.79	0.002
		>150 mg/kg	6	-3.06 [-4.98, -1.15]	90.9	

By conducting subgroup analyses, statistically significant between-group differences were observed in the following outcomes, suggesting possible sources of heterogeneity: serum FSH, testosterone, and LH levels, body weight, sperm count, serum and tissue MDA activity, tissue GSH, SOD, and blood sugar levels.

## 4 Discussion

Using data from 24 animal studies, we found that melatonin can markedly improve male infertility in rodents with metabolic diseases. Melatonin significantly enhanced sperm count and motility, decreased abnormal morphology, and reduced the thickness of the seminiferous tubular basement membrane. Besides, melatonin increased seminiferous epithelial height and tubular diameter and improved histopathological tissue score. Mechanistically, we found that melatonin strengthened anti-oxidant defense, ameliorated oxidative stress, and prevented apoptosis of gonadal cells. Although male rodents who received melatonin had higher levels of testosterone, FSH, and LH, the results did not reach the significance threshold in the main analysis. However, sensitivity analysis indicated that rodents receiving a cumulative dose of melatonin >210 mg/kg had significantly higher levels of LH and FSH, compared with those receiving a cumulative dose of melatonin ≤210 mg/kg. Furthermore, after treatment with melatonin, high-fat diet rodents had significantly higher levels of testosterone, compared with rodents undergoing streptozotocin-induced diabetes. Similarly, treatment with melatonin led to a significant increase in testosterone levels in diabetic rodents, compared with obese rodents.

### 4.1 Histopathology

Awad et al. reported that the seminal level of melatonin is lower than its serum concentration in males with fertile normozoospermia, oligo asthenozoospermia, and non-obstructive azoospermia (50). They observed that there is a significant and positive correlation between the serum level and seminal levels of melatonin (50). Furthermore, all infertile groups had lower serum and seminal levels of melatonin compared with fertile normozoospermic men, and there were strong correlations between sperm motility and serum and seminal levels of melatonin (50). Consistently, a Chinese cross-sectional study with 970 outpatients from a reproductive medicine center reported that men with poor sleep quality have lower total motility, progressive motility, concentration, total count, and normal sperm morphology (51). Interestingly, a recent clinical trial reported that compared with the placebo group, the use of melatonin for 3 months after sub-inguinal varicocelectomy significantly improved several parameters of semen analysis, including sperm concentration, motility, and normal morphology, showing the protective effects of melatonin (52).

Metabolic diseases, such as diabetes, can distort the germinal layers, degenerate seminiferous tubules and interstitial tissues, decrease the diameter of seminiferous tubules, and reduce germinal epithelium height (53–55). Based on our findings, melatonin improved histopathological tissue score and increased seminiferous epithelial height and tubular diameter. Efficient spermatogenesis relies on the complex interaction between Sertoli cells and germ cells in seminiferous tubules (56); therefore, by preserving the normal structure of seminiferous tubules in metabolic diseases, melatonin can potentially provide the prerequisites for spermatogenesis.

### 4.2 Reproductive hormone levels

Most of the previous studies reported that melatonin activates the HPG axis, and increases the serum level of gonadotropin-releasing hormone (GnRH), LH, FSH, and testosterone (17); however, some studies reported the opposite. For instance, Oliveira et al. reported that melatonin downregulates neurokinin B and kisspeptin, two stimulators of GnRH release, in male diabetic rats (57, 58). They claimed that pinealectomy can upregulate GnRh and potentiate the HPG axis in adult rats (59). On the other hand, melatonin is a metabolic regulator of Leydig cells and protects them against harmful stimuli such as metabolic stress (28, 60). Melatonin can prevent apoptosis of Leydig cells and promote the expression of testosterone synthesis-related genes such as steroidogenic acute regulatory protein (*StAR*), transcription factor GATA-4 (*Gata4*), and steroidogenic factor 1 (*SF1*) in Leydig cells (61); thus, melatonin can restore testosterone production by Leydig cells in stress conditions. The pooled data from all studies revealed that treatment with melatonin did not significantly alter the serum levels of testosterone, LH, and FSH in rodents with metabolic diseases, but subgroup group analysis revealed that the cumulative dose of melatonin and the type of metabolic disease should be considered when interpreting the effect of melatonin on sex hormones. Herein, higher cumulative doses (>210 mg/kg) of melatonin markedly increased the serum levels of LH and FSH in rodents with metabolic diseases, while lower doses of melatonin were less effective. Similarly, melatonin more effectively increased the serum levels of testosterone in diabetic rodents compared with obese rodents.

### 4.3 Oxidative stress

Previously, several studies have revealed that metabolic diseases such as obesity, diabetes, dyslipidemia, hypothyroidism, and hyperthyroidism can lead to excessive oxidative stress in the testes, while melatonin can vigorously potentiate endogenous anti-oxidant defense by upregulating SOD, GSH, and GPx (30, 37, 49, 52, 62, 63). Melatonin can increase the testicular expression of nuclear factor erythroid 2–related factor 2 (Nrf2), which is a main transcription factor for many anti-oxidant genes and strengthens the anti-oxidant defense (64). Our meta-analysis showed that melatonin can markedly decrease the serum and tissue levels of MDA and significantly upregulate GSH; however, results were non-significant for other anti-oxidants such as SOD, CAT, and GPx. However, subgroup analysis revealed that melatonin could significantly upregulate them in some subgroups of rodents. For example, melatonin significantly increased SOD activity in alloxan-treated rodents compared with high-fat diet-fed rodents. In addition to oxidative stress, melatonin significantly mitigated the inflammatory response and suppressed the expression of inflammatory cytokines, such as tumor necrosis factor α (TNF-α) and interleukin 1β (IL1β), in the testicular tissue of high-fat diet rats (42, 62).

### 4.4 Effects on apoptosis

Moreover, melatonin can regulate the metabolism of Sertoli cells (65). Herein, it has been shown that melatonin can increase glucose uptake of Sertoli cells by upregulating glucose transporter 1 (GLUT1) and simultaneously enhance their lactate production ability (65). As lactate is a source of energy for germ cells and possesses anti-apoptotic properties, it can immensely improve male infertility (65). Besides, it was observed that the high glycemic status in diabetic or hyperlipidemic mice can induce severe endoplasmic reticulum (ER) stress and apoptosis in Sertoli cells, while treatment with melatonin reverses these alterations (28, 29). Similarly, using a mice model of palmitic acid-induced lipotoxicity, Xu et al. unveiled that melatonin attenuates ER stress, mitochondrial dysfunction, oxidative stress, and apoptosis in spermatogonial stem cells and enhances sirtuin 1 (SIRT1)-mediated p53 deacetylation, which allows G2/M transition and allows cell cycle progression (52). Metabolic perturbation suppressed AMPK/SIRT1 in the testicular tissue of diabetic mice and high-fat diet-fed mice, while treatment with melatonin markedly enhanced the AMP-activated protein kinase (AMPK)/SIRT1 pathway in their testis (30, 64). Consistently, SIRT1 knockdown abolished the protective effects of melatonin on the detrimental effects of high glucose concentration on Leydig cells (64). Previously, it has been demonstrated that SIRT1 is a master regulator of metabolism and pivotal for spermatogenesis and spermatozoa differentiation (66). Mice with SIRT1 deletion had smaller testis, reduced number of spermatozoa, higher percentages of abnormal spermatozoa, and decreased fertility (66). In addition, melatonin treatment was shown to ameliorate impaired autophagy, mitophagy, and mitochondrial biogenesis in Leydig cells exposed to high glucose concentrations (64).

Our meta-analysis demonstrated that melatonin not only improves dyslipidemia-induced inflammatory response in the testicular tissue but also significantly reduces the serum levels of total cholesterol and LDL. Additionally, our meta-analysis revealed that melatonin partly increases HDL levels and lowers triglyceride levels; however, its effect on the serum level of HDL and triglyceride was not statistically significant.

### 4.5 Strength and limitations

To the best of our knowledge, this is the first systematic review and meta-analysis on the protective effects of melatonin against metabolic disorder-induced injuries to rodents’ testicular tissue. Robust pieces of evidence are provided which may facilitate future research. Although, conclusions should be made with caution as this is a rodent study and the results may not be reproducible in larger animals and human models. Although exploring the sources of heterogeneity is one of the added values of this review, in many cases our efforts failed with high residual heterogeneity. The remaining heterogeneity, which could be due to severe methodological discrepancy among the included studies, may alter the interpretation of the results. Furthermore, when interpreting the results, one must pay attention to the robustness of our results in each outcome and the fact that most of the studies had a high risk of bias. Also, conclusions regarding the effectiveness should consider the fact that none of the included studies have aimed to investigate the adverse events and parallel outcomes during melatonin therapy.

## 5 Conclusion

Our findings indicated that melatonin can mitigate histological damage of male gonadal tissue in metabolic diseases and improve several components of semen analysis such as sperm count, sperm motility, and sperm morphology. Mechanistically, most of the previous animal studies focused on oxidative stress and apoptosis as the underlying mechanisms. These findings strongly encourage prospective clinical studies and randomized controlled trials to assess the safety and efficacy of melatonin for male infertility in male patients with metabolic diseases. Finally, it should be emphasized that further research utilizing standardized models before considering clinical trials is highly needed.

## Data availability statement

The original contributions presented in the study are included in the article/**Supplementary Material**. Further inquiries can be directed to the corresponding author.

## Author contributions

NA and ND conceptualized the study. AS and ND designed the study. ND and AS searched the databases and screened the records for eligibility criteria. ND and FE extracted the data and performed quality assessment. AS performed the analyses and visualized the data. AS and MA provided the draft. NA and SP supervised the word. AS and ND share the first authorship. All authors contributed to the article and approved the submitted version.
