# Supplementary material for: Protective effects of melatonin against oxidative stress induced by metabolic disorders in the male reproductive system: a systematic review and meta-analysis of rodent models
Source: Front Endocrinol (Lausanne). 2023 Jul 5;14:1202560. doi: 10.3389/fendo.2023.1202560 (PMC10354453; doi:10.3389/fendo.2023.1202560)
Supplement: Supplementary file 1 [file DataSheet_1.pdf]

Supplementary material, search strategy

1. **Supplementary material 1**

Search strategy

1.1. Keywords

Melatonin	Testicular function		Mesh terms
Melatonin	"sertoli Cell"	Sterility	Infertility
“N-acetyl-5-methoxy tryptamine”	seminal	Subfertility	Fertility
“n acetyl 5 methoxytryptamine”	fertil*	“Sub-Fertility”	Fertility Agents, Male
"Mela-T"	epididymis	Aspermia	Testis
Melatol	"Vas deferens"	Fecundability	Epididymis
Melatonex	testes	Fecundity	Vas deferens
Melovine	testis	Subfecundity	Spermatogenesis
Regulin	testicular	Testicles	Testosterone
“Night NEXT Rest”	sterility	Testicle	Seminiferous Tubules
Circadin	sperm	“Ductus Deferens”	Seminiferous Epithelium
	spermat*	Fertility AND Agents AND Male	Spermatozoa
	Semen	Spermiogenesis	Semen Analysis
	reproduction	Semen AND Analysis	Genitalia, Male
	preconception	“17-beta-Hydroxy-4-Androsten-3-one”	Melatonin
	testosterone	“17 beta Hydroxy 4 Androsten 3 one”	
	"leydig cell"	"Seminiferous Tubule"	
	seminiferous	"Seminiferous Tubules"	
	oligospermia	Seminiferous AND epithelium	
	azoospermia	Seminiferous AND epitheliums	
	astenozoospermia	Semen AND Analyses	
	infertil*	Semen AND Quality	
	Hypospermatogenes*	Sperm AND Quality	

	Oligoasthenoteratozoospermia*	Semen AND Qualities	
	Oligozoospermia	Sperm AND Qualities	
	Genital* AND Male		
	Reproductive AND Male		

1.2. PubMed

(Melatonin[tiab] OR "N-acetyl-5-methoxy tryptamine"[tiab] OR "n acetyl 5 methoxytryptamine"[tiab] OR "Mela-T"[tiab] OR Melatol[tiab] OR Melatonex[tiab] OR Melovine[tiab] OR Regulin[tiab] OR "Night NEXT Rest"[tiab] OR Circadin[tiab]) AND ("sertoli Cell"[tiab] OR (sertoli[tiab] AND cell*[tiab]) OR seminal[tiab] OR fertil*[tiab] OR epididymis[tiab] OR "vas deferens"[tiab] OR (vas[tiab] AND deferens[tiab]) OR testes[tiab] OR testis[tiab] OR testicular [tiab] OR sterility[tiab] OR sperm*[tiab] OR Semen[tiab] OR reproduction[tiab] OR preconception[tiab] OR testosterone[tiab] OR "leydig cell"[tiab] OR (leydig[tiab] AND cell*[tiab]) OR "Seminiferous Tubule"[tiab] OR "Seminiferous epithelium"[tiab] OR (Seminiferous[tiab] AND Tubule*[tiab]) OR (Seminiferous[tiab] AND epithelium*[tiab]) OR oligospermia[tiab] OR hypospermatogenes*[tiab] OR Oligoasthenoteratozoospermia*[tiab] OR Oligozoospermia[tiab] OR azoospermia[tiab] OR astenozoospermia[tiab] OR infertil*[tiab] OR subfertility[tiab] OR "Sub-fertility"[tiab] OR fertil*[tiab] OR fecundability[tiab] OR fecundity[tiab] OR subfecundity[tiab] OR Aspermia[tiab] OR testicle*[tiab] OR (Ductus[tiab] AND Deferens[tiab]) OR "Ductus Deferens"[tiab] OR ("male fertility"[tiab] AND Agents[tiab]) OR "17-beta-Hydroxy-4-Androsten-3-one"[tiab] OR "17 beta Hydroxy 4 Androsten 3 one"[tiab] OR (semen[tiab] AND analys*[tiab]) OR (semen[tiab] AND qualit*[tiab]) OR (sperm[tiab] AND qualit*[tiab]) OR (Genital*[tiab] AND Male[tiab]) OR (Reproducti*[tiab] AND Male[tiab])) OR ((Melatonin[Mesh]) AND ((“Infertility”[Mesh]) OR (“Fertility”[Mesh]) OR (“Fertility Agents, Male”[Mesh]) OR (“Testis”[Mesh]) OR (“Epididymis”[Mesh]) OR (“Vas deferens”[Mesh]) OR (“Spermatogenesis”[Mesh]) OR (“Testosterone”[Mesh]) OR (“Seminiferous Tubules”[Mesh]) OR (“Seminiferous Epithelium”[Mesh]) OR (“Spermatozoa”[Mesh]) OR (“Semen Analysis”[Mesh]) OR (“Genitalia, Male”[Mesh])) AND (1965/1/1:2022/9/9[dp])

Results: 1375

1.3. Scopus

TITLE-ABS-KEY(Melatonin OR "N-acetyl-5-methoxy tryptamine" OR "n acetyl 5 methoxytryptamine" OR "Mela-T" OR Melatol OR Melatonex OR Melovine OR Regulin OR "Night NEXT Rest" OR Circadin) AND TITLE-ABS-KEY("sertoli Cell" OR (sertoli AND cell*) OR seminal OR fertil* OR epididymis OR "vas deferens" OR (vas AND deferens) OR testes OR testis OR testicular OR sterility OR sperm* OR Semen OR reproduction OR preconception OR testosterone OR "leydig cell" OR (leydig AND cell*) OR "Seminiferous Tubule" OR "Seminiferous epithelium" OR (Seminiferous AND Tubule*) OR (Seminiferous AND epithelium*) OR oligospermia OR hypospermatogenes* OR Oligoasthenoteratozoospermia* OR Oligozoospermia OR azoospermia OR astenozoospermia OR infertil* OR subfertility OR "Sub-fertility" OR fertil* OR fecundability OR fecundity OR subfecundity OR Aspermia OR testicle* OR (Ductus AND Deferens) OR "Ductus Deferens" OR ("male fertility" AND Agents) OR "17-

beta-Hydroxy-4-Androsten-3-one" OR "17 beta Hydroxy 4 Androsten 3 one" OR (semen AND analys*) OR (semen AND qualit*) OR (sperm AND qualit*) OR (Genital* AND Male) OR (Reproducti* AND Male)) AND PUBYEAR > 1965 AND PUBYEAR < 2023

Results: 4826

1.4. Web of science

TS=(Melatonin OR "N-acetyl-5-methoxy tryptamine" OR "n acetyl 5 methoxytryptamine" OR "Mela-T" OR Melatol OR Melatonex OR Melovine OR Regulin OR "Night NEXT Rest" OR Circadin) AND TS=("sertoli Cell" OR (sertoli AND cell*) OR seminal OR fertil* OR epididymis OR "vas deferens" OR (vas AND deferens) OR testes OR testis OR testicular OR sterility OR sperm* OR Semen OR reproduction OR preconception OR testosterone OR "leydig cell" OR (leydig AND cell*) OR "Seminiferous Tubule" OR "Seminiferous epithelium" OR (Seminiferous AND Tubule*) OR (Seminiferous AND epithelium*) OR oligospermia OR hypospermatogenes* OR Oligoasthenoteratozoospermia* OR Oligozoospermia OR azoospermia OR astenozoospermia OR infertil* OR subfertility OR "Sub-fertility" OR fertil* OR fecundability OR fecundity OR subfecundity OR Aspermia OR testicle* OR (Ductus AND Deferens) OR "Ductus Deferens" OR ("male fertility" AND Agents) OR "17-beta-Hydroxy-4-Androsten-3-one" OR "17 beta Hydroxy 4 Androsten 3 one" OR (semen AND analys*) OR (semen AND qualit*) OR (sperm AND qualit*) OR (Genital* AND Male) OR (Reproducti* AND Male)) AND DOP=(1965-01-01/2022-09-09)

Results: 3838
