# Supplementary material for: Protective effects of melatonin against oxidative stress induced by metabolic disorders in the male reproductive system: a systematic review and meta-analysis of rodent models
Source: Front Endocrinol (Lausanne). 2023 Jul 5;14:1202560. doi: 10.3389/fendo.2023.1202560 (PMC10354453; doi:10.3389/fendo.2023.1202560)
Supplement: Supplementary file 2 [file DataSheet_2.pdf]

Supplementary material, detailed table

Study name	Mechanism of injury induction	Duration of injury	Dose of injury	Cumulative dose of injury	Route of injury	Duration of melatonin therapy	Dose of melatonin therapy	Cumulative dose of melatonin therapy	Route of melatonin therapy	Timing of intervention*
**Du 2018 (1)**	Streptozotocin	1 day	100 mg/kg	100 mg/kg	IP	14 and 56 days	10 mg/kg	140 and 560 mg/kg	Oral	After
**Guneli 2008 (2)**	Streptozotocin	1 day	45 mg/kg	45 mg/kg	IP	5 days	10 mg/kg	50 mg/kg	IP	After
**Sahan 2020 (3)**	Streptozotocin	1 day	60 mg/kg	60 mg/kg	IP	70 days	10 mg/kg	700 mg/kg	IP	After
**Oliveira 2015 (4)**	Streptozotocin	1 day	120 mg/kg	120 mg/kg	IP	14 days	0.2 mg/kg	2.8 mg/kg	Oral	After
**Saidi 2022 (5)**	High-fat diet	84 days	NA	NA	NA	84 days	4 mg/kg	336 mg/kg	Oral	After
**Akman 2015 (6)**	Alloxan	2 days	120 mg/kg	240 mg/kg	IP	15 and 45 days	10 mg/kg	150 and 450 mg/kg	IP	After
**Zhang 2012 (7)**	High-fat diet	84 days	NA	NA	NA	84 days	1 mg/kg	84 mg/kg	Oral	After
**Khalil 2021 (8)**	High-fat diet	35 days	23.4 kJ/g	819 kJ/g	NA	35 days	10 mg/kg	350 mg/kg	IP	After
**Mohamed 2017 (9)**	Thyroxin<br>Carbimazole	15 days	0.2 mg/kg<br>1.35 mg/kg	3 mg/kg<br>27 mg/kg	Oral	15 days	2.5 mg/kg	37.5 mg/kg	Oral	After
**Oladele 2021 (10)**	High-fat diet	84 days	NA	NA	Oral	84 days	4 mg/kg	336 mg/kg	Oral	After
**Mogulkoc 2005 (11)**	Levothyroxine	21 days	0.3 mg/kg	6.3 mg/kg	IP	21 days	3 mg/kg	63 mg/kg	IP	After
**Atilgan 2013 (12)**	High-fat diet	56 days	NA	NA	NA	1 day	50 mg/kg	50 mg/kg	IP	After
**Aslankoc 2019 (13)**	High-fructose diet	70 days	NA	NA	Oral	42 days	10 mg/kg	420 mg/kg	Oral	After
**Nasiraei-Moghadam 2015 (14)**	One-third of the normal daily food ration	14 days	7.5 g	105 g	NA	14 days	5 mg/kg	70 mg/kg	IP	After
**Chen 2019 (15)**	High-fat diet	84 days	NA	NA	NA	84 days	1 mg/kg	84 mg/kg	Oral	After
**Almabhouh 2016 (16)**	Leptin	42 days	60 µg/kg	2520 µg/kg	IP	42 days	10 and 20 mg/kg	420 and 480 mg/kg	Oral	After
**Almabhouh 2018 (17)**	Leptin	42 days	60 µg/kg	2520 µg/kg	IP	42 days	10 and 20 mg/kg	420 and 480 mg/kg	Oral	After
**Alves 2020 (18)**	Streptozotocin	1 day	60 mg/kg	60 mg/kg	IP	20 days	10 mg/kg	200 mg/kg	Oral	After
**Ramadan 2020 (19)**	Levothyroxine	21 days	0.2 mg/kg	4.2 mg/kg	IP	21 days	1, 5, and 10 mg/kg	21, 105, and 210 mg/kg	IP	After
**Gobbo 2015 (20)**	Streptozotocin	1 day	4.5 mg/100g	4.5 mg/100g	IP	63 and 112 days	10 µg/kg	630 and 1120 µg/kg	Oral	After
**Armagan 2006 (21)**	Streptozotocin	1 day	35 mg/kg	35 mg/kg	IP	56 days	10 mg/kg	560 mg/kg	IP	After
**Hassen 2007 (22)**	Alloxan	1 day	60 mg/kg	60 mg/kg	IP	42 days	2.5 mg/kg	105 mg/kg	Oral	After
**Da Costa 2016 (23)**	Streptozotocin	1 day	4.5 mg/100g	4.5 mg/100g	IP	70 and 119 days	10 µg/kg	70 and 1190 µg/kg	Oral	After
**Buzkurt 2019 (24)**	Streptozotocin	1 day	50 mg/kg	50 mg/kg	IP	7 days	20 mg/kg	140 mg/kg	IP	After

Supplementary table: The detailed characteristics of the injury inductions and melatonin therapy.
*** Relative to induction of injury**

1. Du Z, Xu S, Hu S, Yang H, Zhou Z, Sidhu K, et al. Melatonin attenuates detrimental effects of diabetes on the niche of mouse spermatogonial stem cells by maintaining Leydig cells. Cell Death Dis. 2018;9(10):968.
2. Guneli E, Tugyan K, Ozturk H, Gumustekin M, Cilaker S, Uysal N. Effect of melatonin on testicular damage in streptozotocin-induced diabetes rats. Eur Surg Res. 2008;40(4):354-60.
3. Sahan A, Akbal C, Tavukcu HH, Cevik O, Cetinel S, Sekerci CA, et al. Melatonin prevents deterioration of erectile function in streptozotocin-induced diabetic rats via sirtuin-1 expression. Andrologia. 2020;52(9):e13639.
4. Oliveira ACd, Andreotti S, Sertie RAL, Campana AB, de Proença ARG, Vasconcelos RP, et al. Combined treatment with melatonin and insulin improves glycemic control, white adipose tissue metabolism and reproductive axis of diabetic male rats. Life Sciences. 2018;199:158-66.
5. Saidi AO, Akintayo CO, Atuma CL, Mahmud H, Sabinari IW, Oniyide AA, et al. Melatonin supplementation preserves testicular function by attenuating lactate production and oxidative stress in high fat diet-induced obese rat model. Theriogenology. 2022;187:19-26.
6. Akman O, Sengul E, ÖZkanlar S, Serpil C, Koç A, Karadeniz A, et al. The Melatonin Attenuates Alloxan Induced Post-Diabetic Testicular Damage and Oxidative Effects in Rats2015.

Supplementary material, detailed table

7. Zhang K, Lv Z, Jia X, Huang D. Melatonin prevents testicular damage in hyperlipidaemic mice. Andrologia. 2012;44(4):230-6.
8. Khalil SS, Aziz JA, Ismail KA, El-Malkey NF. Comparative protective effects of N-acetylcysteine and melatonin against obesity-induced testicular dysfunction in rats. Can J Physiol Pharmacol. 2021;99(7):708-19.
9. Mohamed H, Bushra R. Effects of Simultaneous Melatonin Administration on the Testis of the Experimentally Induced Hyper- and Hypothyroidism in the Adult Male Albino Rat. Egyptian Journal of Histology. 2017;40:52-67.
10. Oladele CA, Akintayo CO, Badejogbin OC, Oniyide AA, Omoaghe AO, Agunbiade TB, et al. Melatonin ameliorates endocrine dysfunction and defective sperm integrity associated with high-fat diet-induced obesity in male Wistar rats. Andrologia. 2022;54(1):e14242.
11. Mogulkoc R, Baltaci AK, Oztekin Menevse E, Aydin L, Tuncer I. Hyperthyroidism causes lipid peroxidation in kidney and testis tissues of rats: Protective role of melatonin. Neuro endocrinology letters. 2005;26:806-10.
12. Atilgan D, Parlaktas BS, Uluocak N, Erdemir F, Kilic S, Erkorkmaz U, et al. Weight loss and melatonin reduce obesity-induced oxidative damage in rat testis. Adv Urol. 2013;2013:836121.
13. Aslankoc R, Ozmen O. The effects of high-fructose corn syrup consumption on testis physiopathology-The ameliorative role of melatonin. Andrologia. 2019;51(8):e13327.
14. Nasiraei-Moghadam S, Parivar K, Ahmadiani A, Movahhedin M, Mahdavi MR. Food deprivation and social inequality may lead to oxidative damage: a study on the preventive role of melatonin in the male rat reproductive system. Reprod Fertil Dev. 2015.
15. Chen C, Ling MY, Lin FH, Xu L, Lv ZM. Melatonin appears to protect against steroidogenic collapse in both mice fed with high-fat diet and H(2) O(2) -treated TM3 cells. Andrologia. 2019;51(8):e13323.
16. Almabhouh FA, Osman K, Ibrahim SF, Gupalo S, Gnanou J, Ibrahim E, et al. Melatonin ameliorates the adverse effects of leptin on sperm. Asian J Androl. 2017;19(6):647-54.
17. Almabhouh FA, Singh HJ. Adverse effects of leptin on histone-to-protamine transition during spermatogenesis are prevented by melatonin in Sprague-Dawley rats. Andrologia. 2018;50(1).
18. Alves É R, Ferreira CGM, Silva MVD, Vieira Filho LD, Silva Junior VAD, Melo IMF, et al. Protective action of melatonin on diabetic rat testis at cellular, hormonal and immunohistochemical levels. Acta Histochem. 2020;122(5):151559.
19. Ramadan HM, Taha NA, Ahmed HH. Melatonin enhances antioxidant defenses but could not ameliorate the reproductive disorders in induced hyperthyroidism model in male rats. Environ Sci Pollut Res Int. 2021;28(4):4790-804.
20. Gobbo MG, Costa CF, Silva DG, de Almeida EA, Góes RM. Effect of Melatonin Intake on Oxidative Stress Biomarkers in Male Reproductive Organs of Rats under Experimental Diabetes. Oxid Med Cell Longev. 2015;2015:614579.
21. Armagan A, Uz E, Yilmaz HR, Soyupek S, Oksay T, Ozcelik N. Effects of melatonin on lipid peroxidation and antioxidant enzymes in streptozotocin-induced diabetic rat testis. Asian J Androl. 2006;8(5):595-600.
22. Hassen NS, Omara EA, El Roubi NM. Evaluation of the Influence of each of Melatonin and Chromium against Diabetes-Induced Alteration in the Testis of Albino Rats Using Light and Electron Microscopies. The Egyptian Journal of Hospital Medicine. 2007;27(1):143-62.
23. da Costa CF, Gobbo MG, Taboga SR, Pinto-Fochi ME, Góes RM. Melatonin intake since weaning ameliorates steroidogenic function and sperm motility of streptozotocin-induced diabetic rats. Andrology. 2016;4(3):526-41.
24. Bozkurt A, Karabakan M, Aydin M, Gumustas S, Onk D, Gursul C, et al. Effects of melatonin treatment on the spermatogenesis and serum inflammatory cytokine levels in diabetic rats. 2019;26:12-6.
