# Supplementary material for: Protective effects of melatonin against oxidative stress induced by metabolic disorders in the male reproductive system: a systematic review and meta-analysis of rodent models
Source: Front Endocrinol (Lausanne). 2023 Jul 5;14:1202560. doi: 10.3389/fendo.2023.1202560 (PMC10354453; doi:10.3389/fendo.2023.1202560)
Supplement: Supplementary file 3 [file DataSheet_3.pdf]

Supplementary material, sensitivity analyses

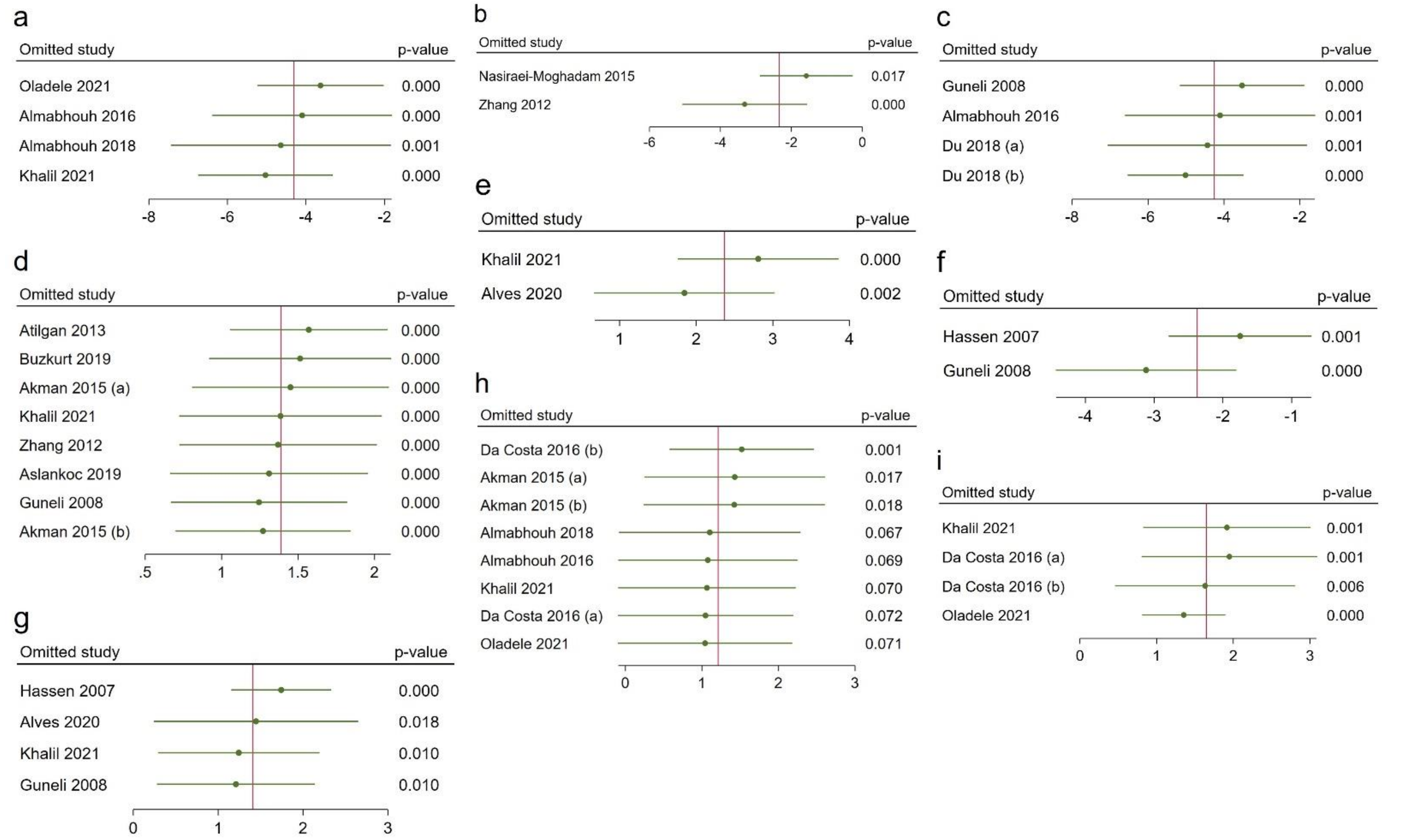

(a) abnormal sperm morphology, (b) apoptotic cells, (c) apoptotic index, (d) Johnsen's testicular biopsy score, (e) seminiferous epithelial height, (f) seminiferous tubular basement membrane thickness, (g) seminiferous tubular diameter, (h) sperm count, (i) sperm motility

Supplementary material, sensitivity analyses

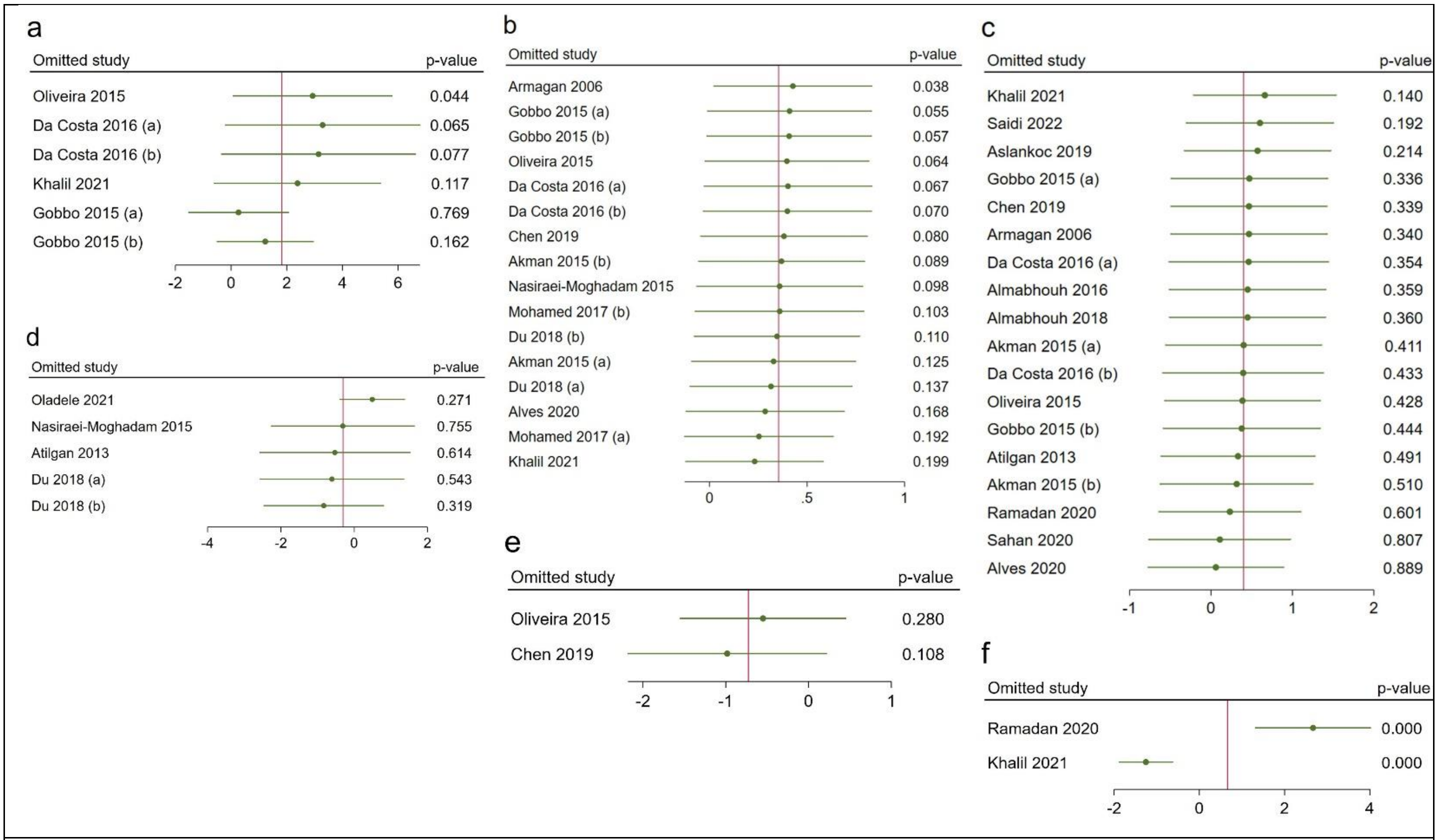

(a) absolute epididymis weight, (b) absolute testis weight, (c) body weight, (d) body weight gain, (e) epididymal adipose tissue weight, (f) relative testis to body weight

Supplementary material, sensitivity analyses

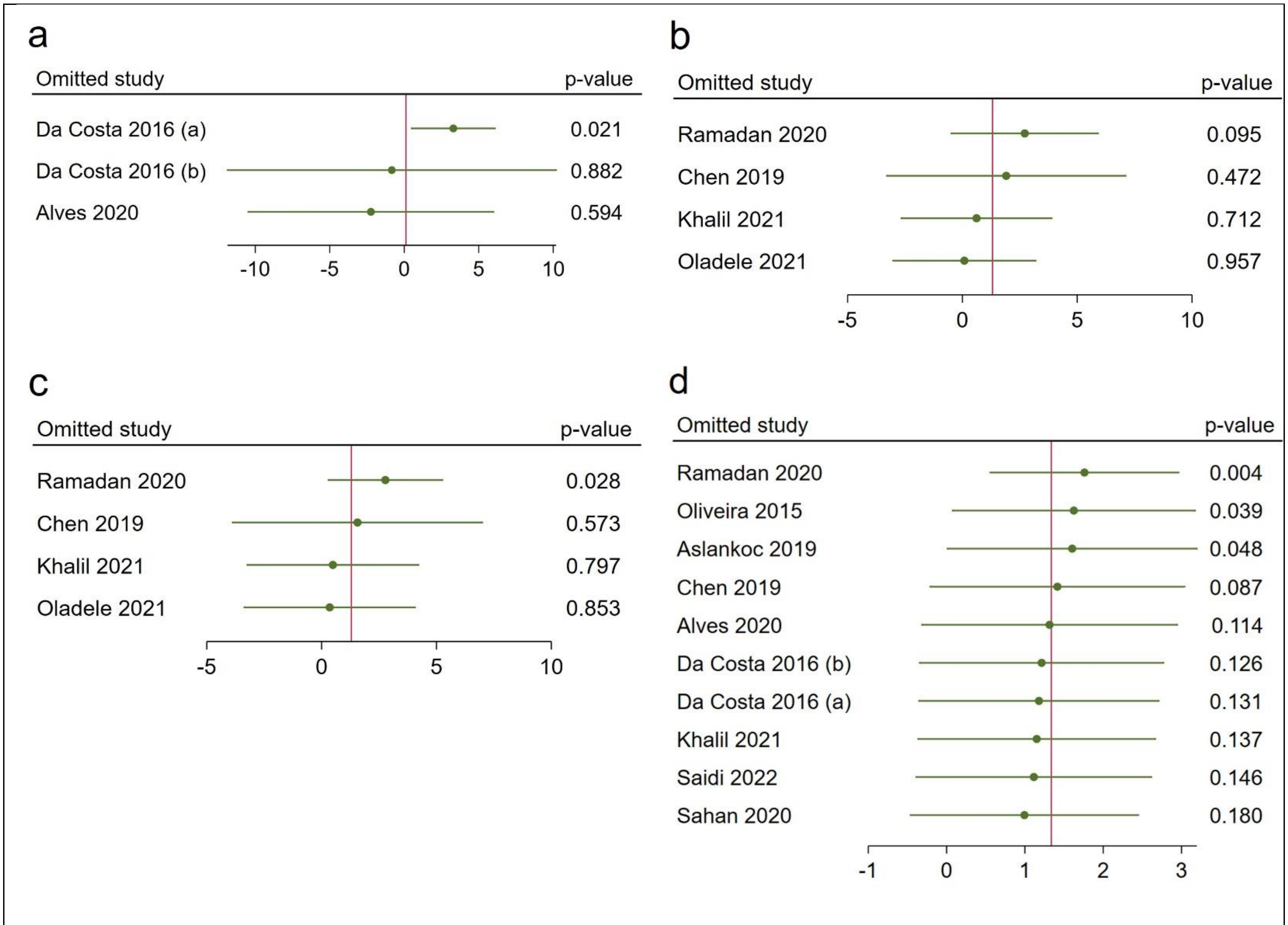

(a) androgen receptor expression, (b) serum FSH, (c) LH, (d) testosterone level

Supplementary material, sensitivity analyses

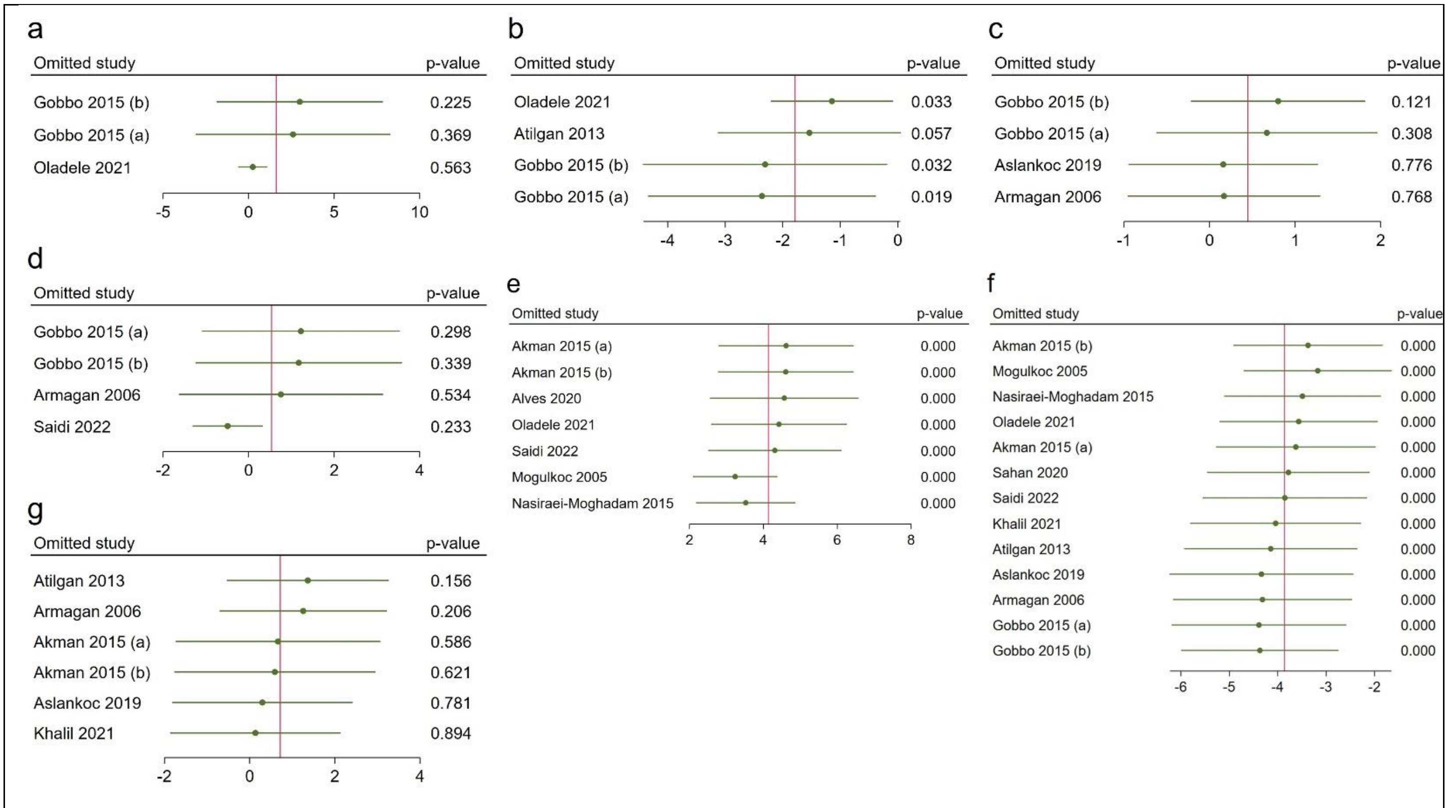

(a) serum GPx activity, (b) serum MDA activity, (c) tissue CAT activity, (d) tissue GPx activity, (e) tissue GSH activity, (f) tissue MDA activity, (g) tissue SOD activity

Supplementary material, sensitivity analyses

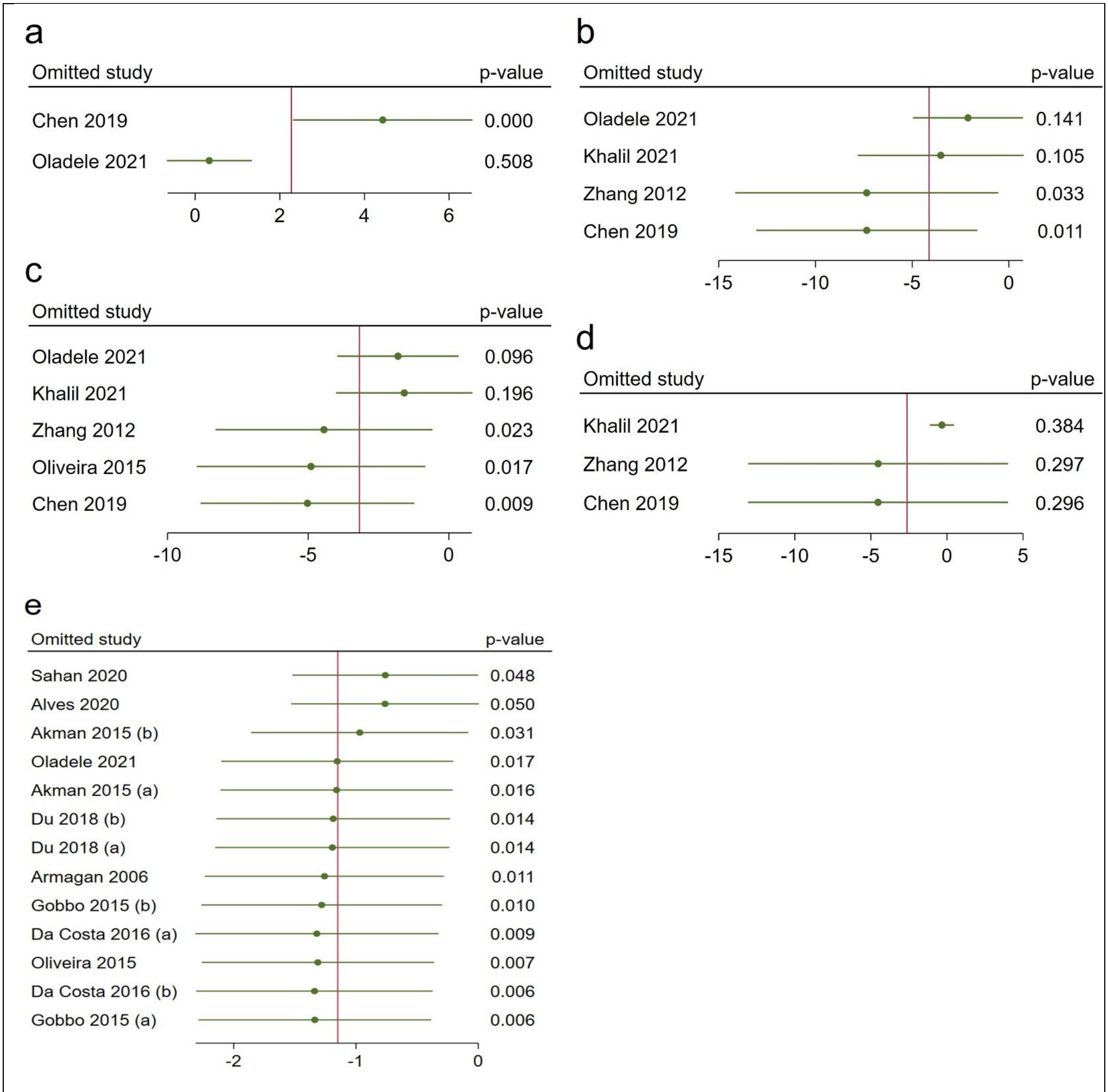

(a) serum HDL, (b) serum LDL, (c) serum total cholesterol, (d) serum triglyceride, (e) blood glucose level
