# Supplementary figures and images for: Protective effects of melatonin against oxidative stress induced by metabolic disorders in the male reproductive system: a systematic review and meta-analysis of rodent models
Source: Front Endocrinol (Lausanne). 2023 Jul 5;14:1202560. doi: 10.3389/fendo.2023.1202560 (PMC10354453; doi:10.3389/fendo.2023.1202560)

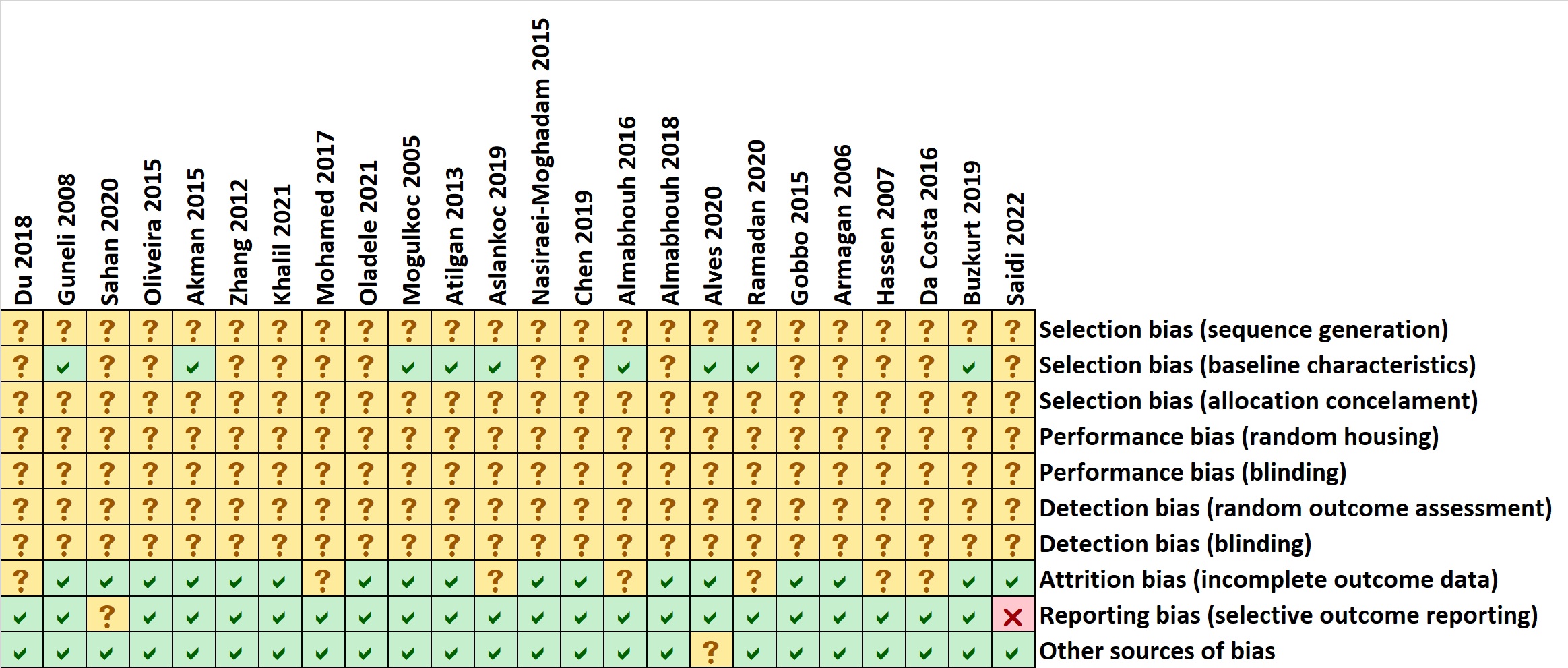

Supplement: Supplementary file 4 [file Image_1.jpeg]

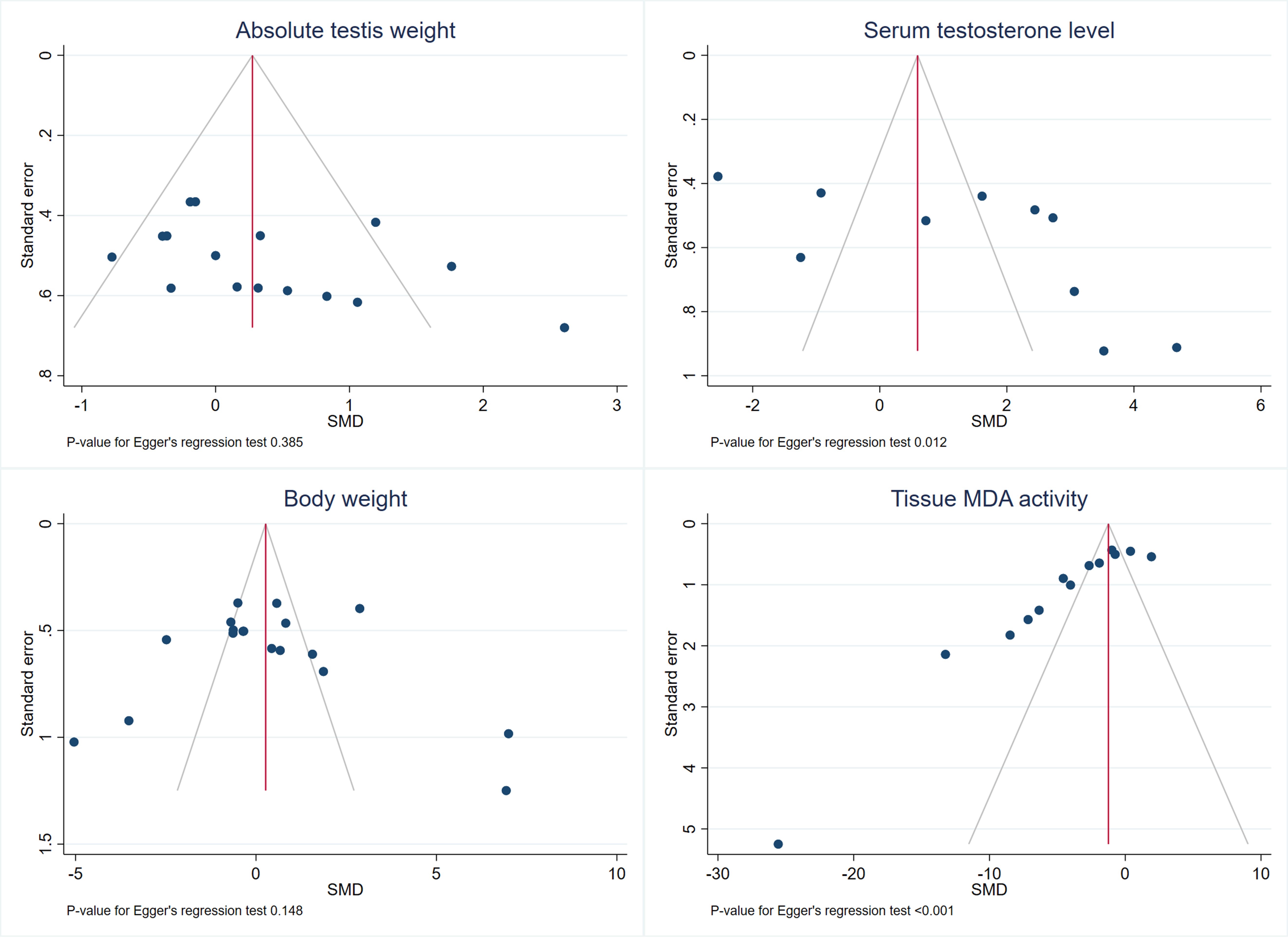

Supplement: Supplementary file 5 [file Image_2.jpeg]

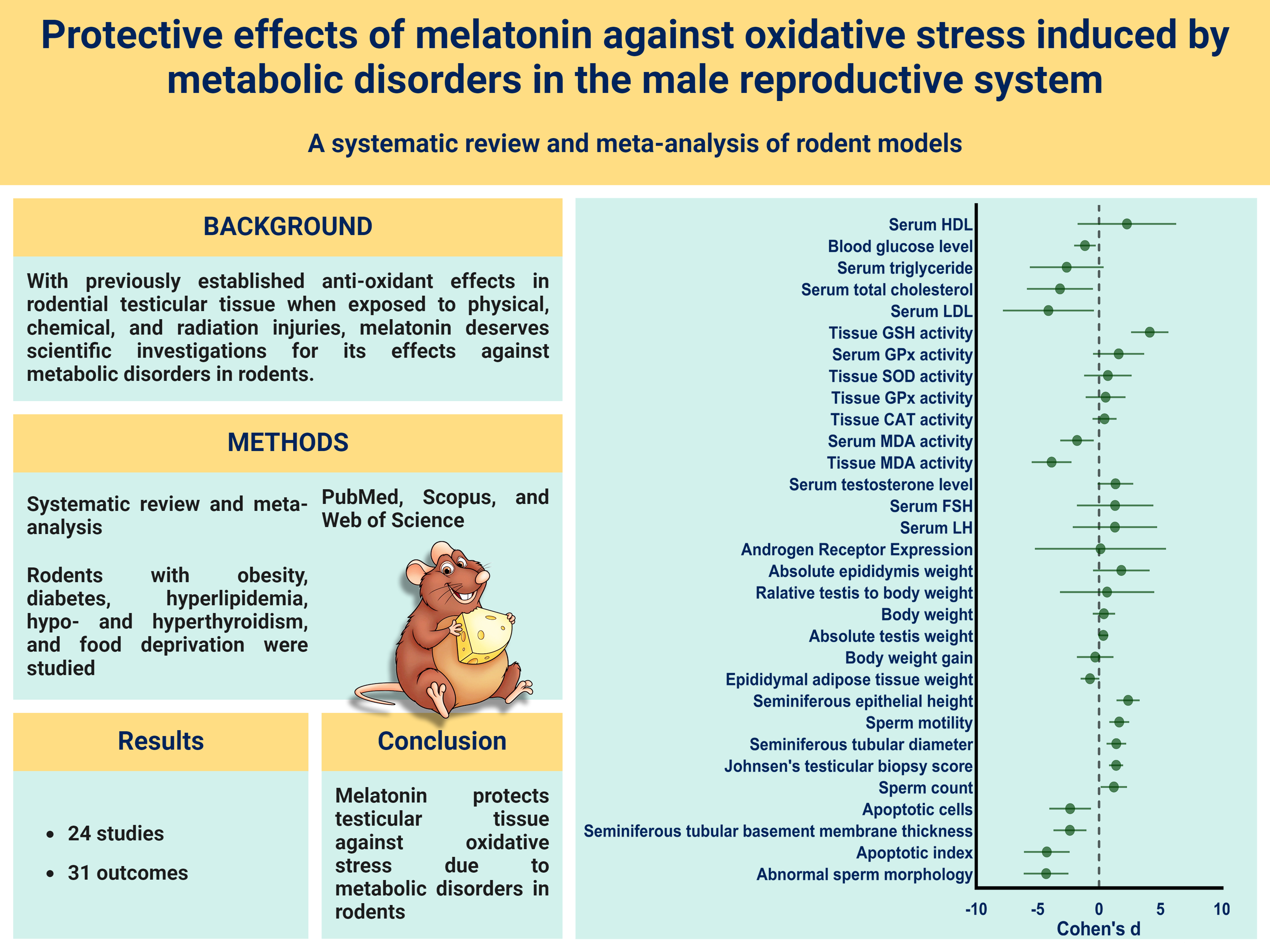

Supplement: Supplementary file 6 [file Image_3.jpeg]
